# *Aegilops crassa* Boiss. repeatome characterized using low-coverage NGS as a source of new FISH markers: Application in phylogenetic studies of the Triticeae

**DOI:** 10.3389/fpls.2022.980764

**Published:** 2022-10-05

**Authors:** Pavel Yu. Kroupin, Ekaterina D. Badaeva, Victoria M. Sokolova, Nadezhda N. Chikida, Maria Kh. Belousova, Sergei A. Surzhikov, Ekaterina A. Nikitina, Alina A. Kocheshkova, Daniil S. Ulyanov, Aleksey S. Ermolaev, Thi Mai Luong Khuat, Olga V. Razumova, Anna I. Yurkina, Gennady I. Karlov, Mikhail G. Divashuk

**Affiliations:** ^1^All-Russia Research Institute of Agricultural Biotechnology, Kurchatov Genomics Centre – ARRIAB, Moscow, Russia; ^2^N.I.Vavilov Institute of General Genetics, Russian Academy of Sciences, Moscow, Russia; ^3^Engelhardt Institute of Molecular Biology, Russian Academy of Sciences, Moscow, Russia; ^4^All-Russian Institute of Plant Genetic Resources (VIR), Department of Wheat Genetic Resources, St. Petersburg, Russia; ^5^Agricultural Genetics Institute, Department of Molecular Biology, Hanoi, Vietnam

**Keywords:** *Aegilops*, shallow whole-genome sequencing, fluorescence *in situ* hybridization, chromosomes, satellite repeats, repeatome

## Abstract

*Aegilops crassa Boiss*. is polyploid grass species that grows in the eastern part of the Fertile Crescent, Afghanistan, and Middle Asia. It consists of tetraploid (4x) and hexaploid (6x) cytotypes (2*n* = 4x = 28, D^1^D^1^X^cr^X^cr^ and 2n = 6x = 42, D^1^D^1^X^cr^X^cr^D^2^D^2^, respectively) that are similar morphologically. Although many *Aegilops* species were used in wheat breeding, the genetic potential of *Ae*. *crassa* has not yet been exploited due to its uncertain origin and significant genome modifications. Tetraploid *Ae*. *crassa* is thought to be the oldest polyploid *Aegilops* species, the subgenomes of which still retain some features of its ancient diploid progenitors. The D^1^ and D^2^ subgenomes of *Ae*. *crassa* were contributed by *Aegilopstauschii* (2*n* = 2x = 14, DD), while the X^cr^ subgenome donor is still unknown. Owing to its ancient origin, *Ae*. *crassa* can serve as model for studying genome evolution. Despite this, *Ae*. *crassa* is poorly studied genetically and no genome sequences were available for this species. We performed low-coverage genome sequencing of 4x and 6x cytotypes of *Ae*. *crassa*, and four *Ae*. *tauschii* accessions belonging to different subspecies; diploid wheatgrass *Thinopyrum bessarabicum* (J^b^ genome), which is phylogenetically close to D (sub)genome species, was taken as an outgroup. Subsequent data analysis using the pipeline RepeatExplorer2 allowed us to characterize the repeatomes of these species and identify several satellite sequences. Some of these sequences are novel, while others are found to be homologous to already known satellite sequences of Triticeae species. The copy number of satellite repeats in genomes of different species and their subgenome (D^1^ or X^cr^) affinity in *Ae*. *crassa* were assessed by means of comparative bioinformatic analysis combined with quantitative PCR (qPCR). Fluorescence *in situ* hybridization (FISH) was performed to map newly identified satellite repeats on chromosomes of common wheat, *Triticum aestivum*, 4x and 6x *Ae*. *crassa*, *Ae*. *tauschii*, and *Th*. *bessarabicum*. The new FISH markers can be used in phylogenetic analyses of the Triticeae for chromosome identification and the assessment of their subgenome affinities and for evaluation of genome/chromosome constitution of wide hybrids or polyploid species.

## Introduction

The genus *Aegilops* L. is closely related to wheat and represents an important gene pool for wheat improvement (Molnár-Láng et al., 2014; Kishii, 2019). Modern taxonomy recognizes 10 diploid and 11 polyploid *Aegilops* species with various genome compositions (Van Slageren, 1994; Kilian et al., 2011). Six major genomic types – S* (Sitopsis section), U (*Ae*. *umbellulata*), C (*Ae*. *markgraphii*), M (*Ae*. *comosa)*, N (*Ae*. *uniaristata*), and D (*Ae*. *tauschii*) - have been distinguished among diploid *Aegilops* species (Kimber and Tsunewaki, 1988); they are thought to have occurred approximately 3 million years ago from hybrid populations of the progenitor of *Ae*. *speltoides* (S genome) × ancient diploid wheat (A genome) *via* the mechanism of homoploid hybrid speciation (Marcussen et al., 2014). Polyploid *Aegilops* emerged from the hybridization of diploid progenitors carrying different genomic types. Despite a broad genome diversity of polyploid *Aegilops*, the D (sub)genome was detected in four, whereas the U in seven species. Depending on the presence of the D or U genome, which are designated as “pivotal,” all polyploid *Aegilops* species are divided into the D genome cluster and U genome cluster (Kimber and Feldman, 1987).

*Ae*. *crassa* Boiss. is a polyploid grass species belonging to the D genome cluster that naturally grows in the eastern part of the Fertile Crescent, from Turkey on the west to Afghanistan and Middle Asia on the east (Kihara, 1957; Van Slageren, 1994; Kilian et al., 2011). *Ae*. *crassa* consists of tetraploid and hexaploid cytotypes [2*n* = 4x = 28, D^1^D^1^X^cr^X^cr^ and 2*n* = 6x = 42, D^1^D^1^X^cr^X^cr^D^2^D^2^ respectively] that are similar morphologically. Based on analysis of variation of repeated DNA sequences, Dubkovsky and Dvořák (1995) suggested that 4x *Ae*. *crassa* is evolutionary an old species, the subgenomes of which were substantially modified during speciation (Kihara, 1949; Kimber and Zhao, 1983; Rayburn and Gill, 1987; Zhang and Dvořák, 1992; Dubkovsky and Dvořák, 1995; Badaeva et al., 1998, 2002, 2021; Dvořák, 1998; Naghavi et al., 2009; Edet et al., 2018). From another side, it may retain some genomic features of ancient diploid ancestors. One of the *Ae*. *crassa* subgenomes, designated D^1^, is related to the D genome of *Ae*. *tauschii* (2*n* = 2x = 14, DD; Kihara, 1949; Kimber and Zhao, 1983; Zhang and Dvořák, 1992; Badaeva et al., 1998, 2002; Edet et al., 2018), which also served as the cytoplasmic genome donor to this tetraploid species (Terachi et al., 1987; Kimber and Tsunewaki, 1988). The D subgenome is also present in two polyploid *Aegilops* species and in hexaploid bread wheat *Triticum aestivum* (2n = 6x = BBAADD). According to molecular analysis of nuclear genome (Dvořák et al., 1998; Luo et al., 2017; Singh et al., 2019) and hybridization pattern of pAs1 probe (Badaeva et al., 1996, 2019a; Zhao et al., 2018; Ebrahimzadegan et al., 2021), the D subgenome of polyploid wheat was inherited from *Ae*. *tauschii* subsp. *strangulata*, while *Ae*. *tauschii* subsp. *tauschii* contributed the D subgenome to polyploid *Aegilops* species *Ae*. *cylindrica* and 6x *Ae*. *crassa* (Badaeva et al., 2002). At the same time, the D^1^ subgenome of *Ae*. *crassa* has probably derived from an ancient *Ae*. *tauschii* and is distinct from both extant *Ae*. *tauschii* subspecies in the distribution of repetitive DNA probes (Rayburn and Gill, 1987; Badaeva et al., 1998, 2002, 2021; Abdolmalaki et al., 2019).

An extinct or still unknown diploid species contributed the second subgenome to *Ae*. *crassa* (Dubkovsky and Dvořák, 1995; Dvořák, 1998; Edet et al., 2018). Kihara (1963) proposed that this subgenome could be inherited from *Ae*. *comosa* and suggested genomic formula DM for tetraploid and DDM for hexaploid *Ae*. *crassa*. Molecular (Zhang and Dvořák, 1992) and meiotic (Kimber and Abu-Bakar, 1981) analyses did not confirm the presence of the M genome in *Ae*. *crassa*. Comparison of the restriction profiles of nuclear repeated nucleotide sequences, RNS (Zhang and Dvořák, 1992; Dubkovsky and Dvořák, 1995; Dvořák, 1998), and DArTseq genotyping (Edet et al., 2018) revealed higher similarity of the X^cr^ subgenome with the S genome of the Sitopsis group, most likely *Ae*. *speltoides*, or with the T genome of *Ae*. *mutica* (Edet et al., 2018). These observations contradict the result of cytogenetic analysis, which showed correspondence to 5S and 45S rDNA patterns of the X^cr^ subgenome chromosomes of *Ae*. *crassa* and the S* genome chromosomes of the diploid Emarginata species, but not with *Ae*. *speltoides* (Badaeva et al., 2002, 2021). Owing to the uncertain origin of the second *Ae*. *crassa* subgenome, Dvořák (1998) proposed the genomic formula DX^cr^ for 4x and DDX^cr^ for 6x cytotype.

RFLP analysis showed that evolution of *Ae*. *crassa* was associated with significant changes in the fraction of repetitive DNA sequences, in particular, five unique fragments emerged on the restriction profiles of *Ae*. *crassa* and its hexaploid derivatives (Zhang and Dvořák, 1992; Dubkovsky and Dvořák, 1995; Dvořák, 1998). Substantial modifications of *Ae*. *crassa* subgenomes compared to its putative diploid progenitors were also revealed using chromosome pairing analysis of intraspecific hybrids (Kihara, 1954, 1963; Zhao and Kimber, 1984), C-banding (Badaeva et al., 1998, 2002), and fluorescence *in situ* hybridization (FISH; Rayburn and Gill, 1987; Badaeva et al., 1998, 2002, 2021; Abdolmalaki et al., 2019).

Despite the significant progress made in genome sequencing, the number of DNA probes employed in FISH analysis of cereal species is still very limited. In addition to pSc119.2 and pAs1 probes that have traditionally been used for chromosome identification and phylogenetic studies of the Triticeae since the middle 80^th^ (Bedbrook et al., 1980; Rayburn and Gill, 1986), several new DNA sequences have been isolated from nuclear DNA and used as probes in FISH analysis of wheat and *Aegilops* as well as of other grass species (Kato et al., 2004, 2011; Komuro et al., 2013; Chen et al., 2019; Xi et al., 2019).

Progress in whole-genome sequencing and bioinformatics pipelines allows obtaining detailed information on the structure of the repeatome. To date, genome assemblies of *Ae*. *tauschii*, *Ae*. *longissima*, *Ae*. *speltoides*, *Ae*. *sharonensis*, and *Ae*. *bicornis* have been obtained (Tiwari et al., 2015; Wang et al., 2021; Avni et al., 2022; Li et al., 2022; Yu et al., 2022). However, the genome of *Ae*. *crassa* has not yet been sequenced, which certainly limits the possibilities of its comprehensive study. From the other side, even unassembled reads can be used to search for new tandem satellite repeats from which chromosomal markers can be developed (Koo et al., 2016; Du et al., 2017; Liu et al., 2018b; Chen et al., 2019; Kroupin et al., 2019b; Nikitina et al., 2020). In particular, comparative genome analysis has been successfully used to obtain specific chromosomal markers for the detection of alien chromosomes in wheat addition lines (Li et al., 2016; Liu et al., 2018a) and for identification of Y subgenome in *Roegneria* (Wu et al., 2021). Koo et al. (2016) mapped satellite repeats identified by flow-sorting and sequencing to chromosome 5M^g^ of *Ae*. *geniculata*. However, in most studies of structural genomic diversity of *Aegilops*, a limited set of “standard” DNA probes based on tandem repeats pTa71, pTa794, pSc119.2, pAs1, and pTa-713, as well as a number of microsatellites, are still being used (Song et al., 2020; Said et al., 2021). Single-gene FISH probes are also employed to compare structural rearrangements of chromosomes in *Aegilops* species (Danilova et al., 2014, 2017; Tiwari et al., 2015; Said et al., 2021). Despite the informativity of the results obtained using single-gene FISH probes, both flow-sorting and creation of cDNA clones remain cost-and labor-consuming procedures. Owing to modern bioinformatics approaches, tandem repeats can be efficiently selected from even shallow whole-genome sequencing data, and new FISH probes can be obtained by either direct labeling of PCR products or by designing labeled oligonucleotides, which facilitates their transfer between different scientific teams (Kroupin et al., 2019a; Lang et al., 2019a; Xi et al., 2020).

In addition to *Ae*. *crassa*, the D subgenome is present in hexaploid wheat and several *Aegilops* species. According to meiotic, cytogenetic, and molecular analyses, the D subgenome of common wheat is not significantly modified relative to the parental (Kimber and Zhao, 1983; Rayburn and Gill, 1987; Dvořák et al., 1998) and therefore can be used for tracing evolutionary changes in the orthologous chromosomes of other Triticeae species. From another side, the wheatgrass (*Thinopyrum*) species are genetically related to D subgenome species of wheat and *Aegilops* (Chen et al., 2001; Guo et al., 2016; Bernhardt et al., 2017, 2020). Introgression of useful genes from wheatgrass to wheat usually occurs between J (wheatgrass) and D (wheat) chromosomes, which might be due to higher homology between them rather than with homoeologues of A or B subgenomes of wheat (Liu et al., 2007; He et al., 2009; Patokar et al., 2015). High syntheny between the J^b^ genome of *Th*. *bessarabicum* and common wheat subgenomes has been detected using a combination of cytogenetic (GISH) and molecular (SNP-mapping) analyses of 12 wheat-*Th*. *bessarabicum* introgression lines (Grewal et al., 2018), indicating that the divergence of wheat and *Th*. *bessarabicum* genomes was not accompanied with large chromosomal rearrangements, but with alterations of the repeated nucleotide sequences. The comparison of copy number variation of transposable elements between polyploid and diploid Triticeae revealed the similarity between J^b^ genome of *Th*. *bessarabicum* and D genome of *Ae*. *tauschii* (Divashuk et al., 2019). Therefore, the comparison of J^b^ and D (sub)genomes in the abundance and chromosomal localization of repeated DNA elements can be informative not only for repeatome and evolutionary studies, but also may have practical application as a source for increasing genetic diversity of wheat.

The aim of our study was to trace evolutionary changes of *Ae*. *crassa* subgenomes (with a special emphasis on the D subgenome) based on a complex approach, which includes low-coverage-sequencing followed by identification of repetitive DNA families using bioinformatics, quantitative assessment of repeats using qPCR, and their physical mapping on chromosomes of *Ae*. *crassa* (4x and 6x) in comparison with diploid *Ae*. *tauschii* (DD), hexaploid common wheat (BBAADD), and diploid wheatgrass *Th*. *bessarabicum* (J^b^J^b^) as an outgroup.

## Materials and methods

### Plant material

The following materials have been used (Table 1). The images of heads, vegetating plants, and spikelets of *Ae*. *crassa* accession K-2485 (4x) and IG 131680 (6x) are shown in Supplementary Figure S1.

**Table 1:** Plant material.

Species	2n	Genome formula	Accessions #	Used in:	Source
*Ae*. *crassa*	28	D^1^D^1^X^cr^X^cr^	AE 742 AE 1649 K-2485	Sequencing, qPCR, FISH FISH FISH	IPK, Gatersleben, Germany VIR, St.-Petersburg, Russia
*Ae*. *crassa*	42	D^1^D^1^X^cr^X^cr^D^2^D^2^	IG 131680	qPCR, FISH	ICARDA, Aleppo, Syria
*Ae*. *tauschii* subsp. *strangulata*	14	DD	K-112	Sequencing, qPCR, FISH	VIR, St.-Petersburg, Russia
*Ae*. *tauschii* subsp. *typica*	14	DD	K-428	Sequencing	VIR, St.-Petersburg, Russia
*Ae*. *tauschii* subsp. *tauschii*	14	DD	K-1619	FISH	VIR, St.-Petersburg, Russia
*Triticum aestivum* cv. Chinese Spring	42	BBAADD	–	FISH	–
*Th*. *bessarabicum*	14	J^b^J^b^	PI 531711	Sequencing, qPCR, FISH	USDA-ARS GRIN

### Sequencing

The fresh young leaves of growing plants were ground in liquid nitrogen, then genomic DNA was extracted using the CTAB protocol (Rogers and Bendich, 1985) and used for whole-genome sequencing, qPCR, and probe preparation for FISH. The quantity and quality of the extracted DNA were checked using a NanoDrop OneC spectrophotometer (Thermo Fisher Scientific) and by electrophoresis in 0.8% agarose gel, respectively. Only genomic DNA samples with OD260/280 value ranging from 1.8 to 2.0 and OD260/230 value ranging from 2.0 to 2.2 were considered as good quality. DNA concentration was measured on a Qubit 4 instrument using Qubit™ dsDNA HS and BR Assay Kits (Thermo Fisher Scientific, Waltham, MA, United States). The shotgun libraries were synthesized using the Swift 2S® Turbo DNA Library Kit (Swift Bioscience, Ann Arbor, MI, USA) according to the manufacturer’s protocol. A test run to check the quality of the libraries was carried out on the MiSeq instrument on MiSeq Reagent Nano Kit v2 (300-cycles). Next, the libraries went through the conversion step and were sequenced on DNBSEQ-G400 on 1 lane. The initial amount of DNA was 25 ng, with the length of fragments around 350 bp and pair-end indexing on Swift 2S Turbo Unique Dual Indexing Kit. The run was performed on the Illumina NextSeq with NextSeq 500/550 Mid Output Kit v2.5 (300 cycles) as described in Illumina protocols for pair-end reads. The length of read was 151 bp, the length of index - 8 bp. The sequencing was performed in Genomed, Ltd. (Moscow, Russia).

### Reads preparation and repeat assembly

Adapter sequence and low-quality reads were removed by bbduk.sh from BBMap package (v38.90, github.com/BioInfoTools/BBMap; Bushnell, 2014) at given parameters: ktrim = r k = 20 mink = 10 hdist = 2 maxns = 0 ftl = 19 ftr = 139 minlen = 100 with default bbmap adapters references. After that, they were additionally trimmed from the 3′-end at interleaved = t ftr = 99 for all the reads prepared for assembly to be the same fixed length. We have controlled the quality of resulting reads by using FastQC (v0.11.5, github.com/s-andrews/FastQC; Andrews, 2010) and sampled 2,000,000 paired interlaced reads from them. Interlaced reads were then forwarded to RepeatExplorer2 (v0.3.8., bitbucket.org/petrnovak/repex_tarean; Novák et al., 2013). RepeatExplorer2 output was parsed using custom scripts (github.com/Stathmin/Scripts-for-RepeatExplorer2-parsing).

### Repeats alignment and identification

Global alignment is not sensitive enough when applied to repeat consensus monomers because of arbitrary selection of their starts. We took another approach, so each repeat consensus was tripled and aligned with consensuses database by blastn from BLAST+ package (v2.9.0, ftp.ncbi.nlm.nih.gov/blast/executables/blast+/2.9.0; Camacho et al., 2009) with –task dc-megablast. All the high-scoring pairs (HSPs) for each aligned pair were analyzed, and total coverages without overlaps of longer sequences by HSPs of shorter ones were calculated with our custom script. We assumed the two repeat consensuses to be related if they produced alignments with e-value <0.05 and their total coverage without overlaps ≥80%. To identify previously known repeats the NCBI Nucleotide database (Coordinators, 2012) was used, and all the repeat-related sequences of Triticeae were downloaded on Oct 18 2021. Each tandem repeat was designated as follows: AC4x_CL##_###nt for 4x *Ae*. *crassa*, AC6x_CL##_###nt for 6x *Ae*. *crassa*, ATs_CL##_###nt for *Ae*.*tauschii* subsp. *strangulata* and ATt_CL##_###nt for subsp. *typica*, TB_CL##_###nt for *Th*. *bessarabicum*, where CL## is the numerical name of a particular repeat cluster and ###nt is the length of its single monomer. For convenience, we used only CL## designations in subsequent qPCR and FISH experiments. The found repeats were submitted to NCBI GenBank system and acquired the IDs ON872662-ON872692.

### Diversity of identified satellites

Acquired repeat consensuses shorter than 200 bp were multiplied until they reached length ≥ 200 bp, which is required for informative mapping with 100 bp paired reads. Samples of 5,000,000 paired reads from each analyzed object, trimmed as described above, were then mapped on acquired repeat consensuses using gsnap (version 2021–12-17, github.com/juliangehring/GMAP-GSNAP; Wu and Nacu, 2010) with flags –max-mismatches 0.2 –min-coverage 40. Produced *.bam mappings were visualized using Jbrowse v1.6.9 (jbrowse.org/jb2/; Buels et al., 2016).

### Repeatome structure comparison

RepeatExplorer2 outputs containing proportions of reads by repeat type were parsed. For all the repeat type categories the cumulative proportions, i.e., including subcategories, were summed up and compared between species. For each category the average proportions, standard deviations, and coefficients of variation were obtained.

### *In silico* identification of X^cr^ subgenome-specific tandem repeats in 4x *Aegilops*. *crassa*

Raw reads of 4x *Ae*. *crassa* were cleaned up from adapter sequences, and reads were truncated till 100 bp from 3′-end using bbduk from BBTools v38.93 toolkit (sourceforge.net/projects/bbmap/). Trimmed reads were mapped on *Ae*. *tauschii* Aet v4.0 genome assembly (Luo et al., 2017) using bwa mem v0.7.17 (github.com/lh3/bwa). For further analysis the reads that perfectly mapped on reference assembly were removed using samtools (Danecek et al., 2021). The resulting 831,000 reads were taken for *de novo* tandem repeats’ identification using RepeatExplorer2 pipeline (Novák et al., 2017). Novel tandem repeats were identified based on previously obtained consensuses sequences from 4x *Ae*. *crassa* using BLAST (Altschul et al., 1990). Primers for identified tandem repeats selected for further estimation of repeat number by qPCR and for probe synthesis for FISH by PCR were designed using Primer3Plus (Untergasser et al., 2012), and their sequences are given in Table 2.

**Table 2:** Repeats and primers used for probe amplification and qPCR analysis.

Repeat	Origin	Primers
CL3	4x *Ae*. *crassa*	F: 5′-ATGCACCATTCAAAGCCACA-3′ R: 5′- ACCATGCCAAGTTTCAACCT-3′
CL8	4x *Ae*. *crassa*	F: 5′-GTTCCTTGACCACGTTGACC-3′ R: 5′-CCATCCCTCTACTCACCCAC-3′
CL18	4x *Ae*. *crassa*	F: 5′-TCCTGTTGTTCTATGTTGACACG-3′ R: 5′-CATGATGGTTTTCGGTAGGG-3′
CL60	4x *Ae*. *crassa*	F: 5′-TCTCCCCTACTCCACCATCA-3′ R: 5′-TCATAAGTGATTGATGCGGGA-3′
CL131	4x *Ae*. *crassa*	F: 5′-CACGGAGGGGATCTTGCTAA-3′ R: 5′-GCCTAACCCTGTAGCGTTTG-3′
CL170	4x *Ae*. *crassa*	F: 5′-AGTTCTCCAAAAGTTCCCTATGA-3′ R: 5′-TGTGACGACCAGATGCTTCA-3′
CL193	4x *Ae*. *crassa*	F: 5′-TGGTCTCACGGCATGGA-3′ R: 5′-ACTCCATGCATGTGTCCA-3′
CL209	4x *Ae*. *crassa*	F: 5′-CAAAGTGGTCGGACATGCG-3′ R: 5′-CCCACGCCAGTTTGCATATT-3′
CL219	4x *Ae*. *crassa*	F: 5′-AAAAGTTGGGTGCATTCCGG-3′ R: 5′-AGTGTTTTCGGGGTTTTCGG-3′
CL228	4x *Ae*. *crassa*	F: 5′-GCAAGAGATCATGTGTGCCA-3′ R: 5′-GGGTCACCGTGTCATAGAGA-3′
CL232	4x *Ae*. *crassa*	F: 5′-CACACGCCCGCATCAC-3′ R: 5′-ATGGCCCTGGTGATGCG-3′
CL239	4x *Ae*. *crassa*	F: 5′-CACAAAGAGAGGTAATATATGGAGACC-3′ R: 5′-CAAACTTTTCTCAAAATAGGTCTCCATAT-3′
CL244	4x *Ae*. *crassa*	F: 5′-AGCGTTCCTGGAAATCTCGG-3′ R: 5′-GTGTGGCTCGTAACACCTGA-3′
CL257	4x *Ae*. *crassa*	F: 5′-ACGTTGCAATCATTTCTATCGGT-3′ R: 5′-ACATATTTCAGTGCTACCAACGA-3′
CL258	4x *Ae*. *crassa*	F: 5′-TGTTGAAATAGAGCGCACCG-3′ R: 5′-CGAAACAATTGCAAACGCGA-3′
CL261	4x *Ae*. *crassa*	F: 5′-CACCCTCACGTTTTCACCAG-3′ R: 5′-TTGTGATTGTTTTGGCGGGT-3′
CL27_232	6x *Ae*. *crassa*	F: CAGGTCGAAGTGTGTGTTGGT R: AAATTAAACGAGCCGAAGGCG
CL2	*Th*. *bessarabicum*	F: 5′-AGCTCGTTCTGACTCACGTT-3′ R: 5′-CGTGCCCTCAAAAATGGATCG-3′
CL148	*Th*. *bessarabicum*	F: 5′-CGATTCAGTAGGAAGCGGGT-3′ R: 5′-AAAATGCGGTCAAAACGGCG-3′

### Real-time quantitative PCR

qPCR using primers developed for repeat monomers (Table 2) was performed in triple technical replication with water as negative control and *VRN1* as a reference gene (Yaakov et al., 2013) according to the protocol described in Kroupin et al. (2019b). The amplification was performed on a CFX Real-Time PCR Detection System (Bio-Rad) and in Real-Time PCR Mix reaction mixture with Eva Green (Syntol Ltd., Moscow, Russia) according to the manufacturer’s protocol. Primers were synthesized at Syntol Ltd. (Moscow, Russia). The primer concentration was 10 ng/μl, and the DNA concentration was 0.4 ng/μl. The amplification program was as follows: pre-incubation for 10 min at 95°C, then 45 cycles: denaturation for 10 s at 95°C; primer annealing for 30 s at 60°C. The relative quantity (RQ) was calculated using Bio-Rad CFX Manager 3.1 software based on the obtained Ct volumes.

### DNA probes for FISH

The following novel repeats identified by means of lowcoverage sequencing followed by bioinformatics analysis were used as FISH probes: (i) derived from 4× *Ae*. *crassa* genome: CL3, CL8 (highly homologous to CL16 found in *Ae*. *tauschii* subsp. *strangulata*), CL18, CL60, CL131 (highly homologous to CL149 found in *Th*. *bessarabicum*), CL170, CL193, CL209, CL219, CL228, CL232, CL239, CL241, CL244, CL257, CL258, CL261 (highly homologous to CL198 found in *Th*. *bessarabicum*); ii) derived from 6x *Ae*. *crassa* genome: CL27_232; iii) derived from *Th*. *bessarabicum* genome: CL2, CL148. The probes were obtained using PCR with primer sets listed in Table 2. PCR amplification was performed in a 15-μl reaction mixture containing approximately 50 ng genomic DNA, 1.5 μl of 10× PCR buffer, 1.5 mM MgCl_2_, 0.2 mM of dNTPs, 0.3 μM of each primer, and 0.5 unit of *Taq* DNA polymerase. The PCR conditions were as follows: an initial denaturation step of 95°C for 5 min, followed by 30 cycles of 94°C for 1 min, annealing at 60°C for 1 min and elongation at 72°C for 1 min with a final extension step at 72°C for 5 min. The obtained amplicons were labeled with either biotin-16-dUTP (CL18, CL60, CL131, CL148, CL170, CL193, CL198, CL219, CL232, CL257, CL258, CL261, CL27_232) or digoxigenin-11-dUTP (CL2, CL3, CL8, CL16, CL18, CL131, CL149, CL170, CL209, CL228, CL232, CL239, CL244) by PCR according to the manufacturer’s instructions (Roche, Germany). Additionally, the probes oligo-pAs1, oligo-pSc119.2, oligo-pTa535 and oligo-pTa71 (Tang et al., 2014), oligo-5S rDNA (Yu et al., 2019), oligo-45 (Tang et al., 2018; Xi et al., 2020), P132 (as homolog to CL241), and P332 derived from *Ae*. *tauschii* (Kroupin et al., 2019b), oligo-713 (Tang et al., 2016), and GAA_n_ labeled at the 5′-end with either 6-carboxyfluorescein (FAM; oligo-pAs1, oligo-pSc119.2, oligo-pTa71, oligo-45, P332, P132, and GAA_n_) or 6-carboxytetramethylrhodamine (TAMRA) or Cy3 (oligo-pAs1, oligo-pTa535, oligo-5S, P332, and oligo-713) were used. For convenience, we further designated the oligo-probes according to the original probe names, i.e., pAs1, pSc119.2, pTa-535, pTa-713, 5S (pTa794), and NOR (pTa71).

### Fluorescence *in situ* hybridization

Chromosomal preparation was carried out according to the protocol published in Badaeva et al. (2017). FISH probes were hybridized to chromosomes of 4x and 6x *Ae*. *crassa*, *Ae*. *tauschii* (subsp. *strangulata* and subsp. *tauschii*), *Th*. *bessarabicum*, and common wheat *T*. *aestivum* cv. Chinese spring according to protocol in Kuznetsova et al. (2019). Biotinilated probes were detected with streptavidin-Cy3 (Vector laboratories, UK) and digoxigenin-labeled probes were detected using anti-digoxigenin-Fluorescein (Roche, Germany). The slides were stained with DAPI (4′,6-diamidino-2-phenylindole) in Vectashield mounting media (Vector laboratories, Peterborough, UK) and analyzed on Leica DM6 B epifluorescence microscope. Selected images were captured with DFC 9000 GTC (Leica), and the slides were then washed in 2× SSC, and the second hybridization was carried out with “standard” DNA probes (pSc119.2, pAs1, pTa-535, pTa-713, and GGA_n_) to allow chromosome identification. Probe combination pAs1 + pSc119.2 was used for wheat and *Aegilops* species and pAs1 + pTa-713 for *Th*. *bessarabicum*. The A subgenome chromosomes of wheat were classified using additional probe combination GAA_n_ and pTa-535 according to Komuro et al. (2013); chromosomes of *Ae*. *tauschii* were classified as suggested by Badaeva et al. (2002, 2019a) and Zhao et al. (2018), *Ae*. *crassa* as in Abdolmalaki et al. (2019), and Badaeva et al. (2021). *Th*. *bessarabicum* chromosomes were classified according to Grewal et al. (2018), Badaeva et al. (2019b), and Chen et al. (2019).

Our previous analyses showed that accessions K-2485 and AE 742 (*Ae*. *crassa*, 4x), K-112 (*Ae*. *tauschii* subsp. *strangulata*), PI 531711 (*Th*. *bessarabicum*), and Chinese Spring have normal karyotypes typical to the respective species (Gill et al., 1991; Badaeva et al., 2019a, 2019b, 2021). At the same time, we have found earlier that hexaploid *Ae*. *crassa*, IG 131680 carries a translocation T1D^1^L:7D^1^L (T10) with interstitial breakpoints in addition to two species-specific translocations, T1 (A^cr^:6X^cr^) and T2 (4D^1^S,F^cr^S; Badaeva et al., 2021).

## Results

### Assemblies’ characterization

Repeat assemblies for single species (“individual” assemblies) were prepared for tetraploid and hexaploid *Ae*. *crassa*, *Th*. *bessarabicum*, *Ae*. *tauschii* subsp. *strangulata*, and subsp. *typica*. The general features of these assemblies are summarized in Supplementary Table S1. In addition, we compiled three comparative assemblies, in which the number of reads for each species were calculated in a direct proportion to ploidy level: 0.5 mln TB + 0.5 mln ATs + 1mln AC4x; 0.5 mln TB + 0.5 mln ATt + 0.5 mln AC4x; 1 mln ATs + 2 mln AC4x. For all repeats extracted from the above assemblies, the extensive summary tables were constructed (Supplementary Table S2). For comparative assembly we have extracted and described clusters containing ≥80% repeats from only one species and, if possible, having no highly similar alignments with clusters from individual assemblies for other species involved in comparative assembly.

### Repeats’ characterization

Altogether, 34 repeats were identified in individual assemblies of *Ae*. *crassa* 4x (11 high confidence, 8 low confidence satellites and 1 LTR) and *Th*. *bessarabicum* (6 high confidence, 6 low confidence satellites and 2 LTRs). The characterization of the identified repeats are shown in Supplementary Tables S3, S4 including consensus sequences, layout of TAREAN graph, estimated proportion of given repeat in the genome, homology to previously found repeats, and repeats found in this study. All repeat consensus sequences (excluding those shorter than 100 bp) together with the putative satellites of 6x *Ae*. *crassa* and *Ae*. *tauschii* have been clustered after all-to-all blast into 19 groups by their identity and total coverage percentage; based on these data we developed 19 probes. Seventeen of these groups contained sequences from *Ae*. *crassa* 4x and were classified into high-probability (№1-№11) and low-probability (№12-№16) repeats, and putative LTR (№17). Additionally, we discriminated an additional group (№18), in which a sequence obtained from 6x *Ae*. *crassa* was not paired with a sequence from 4x *Ae*. *crassa*. Two other satellites (high confidence №19, and low confidence №20) were found in *Th*. *bessarabicum* genome. Based on bioinformatical analysis, all repeats were classified according to copy number as very-low-copy (below 0.29%), low-copy (0.3–0.59%), and common (0.6% and more).

The high-probability repeats, representatives of the groups based on 4x *Ae*. *crassa*:

1. AC4x_CL3_339nt is a common repeat, found in all species analyzed. The density of the TAREAN graph layout produced for the given length of repeat monomer (Supplementary Figure S2a) is high, allowing the assumption that there are self-alike features in the monomer influencing the graph. Subsequent analysis with YASS online tool reveals a non-perfect palindromic region 58–176 with 60% identity and e-value of 9.7e−05. Two similar repeats, Ats_CL11_337nt and Ats_CL51_343nt, were identified in the assembly of *Ae*. *tauschii* subsp. *strangulata* by RepeatExplorer2; the latter differed from the analogous *Ae*. *crassa* monomer mostly in the given palindromic region. AC4x_CL3_339nt was more dissimilar to P335 repeat from *Ae*. *crassa* genome.
2. AC4x_CL131_334nt is a very-low-copy repeat, found in all analyzed species. It is highly similar to P334 found in *Ae*. *tauschii* with 90% identity across the 322 bp alignment and highly similar to pTa-465 (FISH-positive repetitive sequence KC290905.1) with 91% identity across 299 bp.
3. AC4x_CL170_369nt is a very-low-copy repeat found in all species analyzed in this study, which directly corresponds to P369 found in *Ae*. *tauschii*, and is highly similar to tandem repeat sequence 4P6-14 found in *Ae*. *tauschii* (AY249985.1) and ACRI_TR_CL78 satellite sequence found in *Agropyron cristatum* (MG323512.1).
4. AC4x_CL209_316nt is a very-low-copy repeat, found in all species, except for *Th*. *bessarabicum*. It is highly similar to *T*. *aestivum* clone p451 (genomic repeat region AF139201.1), and partially more dissimilar from P320 found in *Ae*. *tauschii*, aligning for 197 bp with 75% identity.
5. AC4x_CL219_319nt is a very-low-copy repeat, found in all species except for *Th*. *bessarabicum* and *Ae*. *tauschii* subsp. *strangulata*. This sequence was found in *Ae*. *tauschii* (AH013688.3) in tandem, but was not annotated.
6. AC4x_CL228_312nt is a very-low-copy repeat not yet annotated at NCBI database and found only in the context of *T*. *aestivum* BAC libraries and assemblies. It is, however, found in our assemblies of both *Ae*. *tauschii* subspecies and 6x *Ae*. *crassa*, but is lacking in *Th*. *bessarabicum*. Mapping of the AC4x_CL228_312nt monomer with trimmed reads from different sources (Supplementary Figure S2c) shows similar mapping profiles between *Ae*. *crassa* 4x and 6x and *Ae*. *tauschii* subsp. *typica* with more notable SNPs in *Ae*. *tauschii* subsp. *strangulata* and lack of given repeat in *Th*. *bessarabicum*. AC4x_CL228_312nt is more dissimilar to P321 found in *Ae*. *tauschii*, although they produce alignment of 242 bp with 92.6% identity.
7. AC4x_CL232_320nt is a very-low-copy repeat, found in all studied species except for *Th*. *bessarabicum*. It is highly similar to *T*. *aestivum* clone, to p451 (genomic repeat region AF139201.1), and partially it shows high similarity with P320, aligning for 276 bp with 96% identity.
8. AC4x_CL241_88nt is a very-low-copy repeat, found across all species. This repeat is 97.72% identical to Oligo-44 and Oligo-3A1 found in *T*. *aestivum* and highly similar to oligonucleotide BSCL184-2 based on tandem repeat BSCL184 (88 bp long) found in *Th*. *bessarabicum* and P132 found in *Ae*. *tauschii*.
9. AC4x_CL244_376nt is a very-low-copy repeat, found in all the species except for *Ae*. *tauschii*. It is highly similar (with 100% identity) to oligos BSCL1-1 and BSCL1-2 based on putative satellite BSCL1 (376 bp long) found in *Th*. *bessarabicum*, and DP4J27982 and DP4J28086 developed based on the sequences of chromosome arm 4J^b^L of *Th*. *bessarabicum*.
10. AC4x_CL257_820nt is a very-low-copy repeat, found exclusively in 4x *Ae*. *crassa* and having no similar annotated sequences.
11. AC4x_CL258_1307nt is a very-low-copy repeat, specific to *Ae*. *crassa*, and is not annotated either in NCBI database or in analyzed literature.

The low-probability repeats, representatives of the groups based on 4x *Ae*. *crassa*:

1. AC4x_CL8_584nt is a common repeat, highly similar to AC6x_CL6_584nt monomer (6x *Ae*. *crassa*) and more dissimilar to monomers ATs_CL16_551nt and ATt_CL17_553nt of both *Ae*. *tauschii* subspecies. According to gsnap mapping (Supplementary Figure S2b), *Th*. *bessarabicum* variant of this repeat differs from the AC4x_CL8_584nt due to the many SNPs. It had no similar annotated sequences either in NCBI database or in analyzed literature.
2. AC4x_CL60_251nt is a low-copy repeat, found exclusively in tetraploid *Ae*. *crassa* and being partially more dissimilar to *Ae*. *bicornis* RAPD-generated marker sequence (AF120172.1), aligning for 200 bp with 78% identity.
3. AC4x_CL193_504nt is a very-low-copy repeat, found in all species except for *Th*. *bessarabicum*. It is somewhat similar to *T*. *aestivum* clone pTa-451 (FISH-positive repetitive sequence KC290912.1) with the alignment of 500 bp with 68% identity.
4. AC4x_CL239_178nt is a very-low-copy repeat, found in all species except for *Ae*. *tauschii*. This sequence is tandemly organized in *Ae*. *tauschii* sequence AH013688.3, but is not annotated either in NCBI database or in analyzed literature.
5. AC4x_CL261_553nt is a very-low-copy repeat, found in all species except for *Ae*. *tauschii*. It is more dissimilar to *T*. *aestivum* clone CentT550 (satellite sequence MN161206.1), aligning across 540 bp with 83.6% identity.

The putative LTR, representative of the groups based on 4x *Ae*. *crassa*:

1. AC4x_CL18_487nt is a common repeat, similar to FAT element.

The high-probability repeats, representative of the groups based on 6x *Ae*. *crassa*:

1. AC6x_CL232_145nt is a low-copy repeat, found only in 6x *Ae*. *crassa* and in both *Ae*. *tauschii* subspecies, being fully identical to P436 found in *Ae*. *tauschii*. The FISH probe developed based on AC6x_CL232_145nt is designated CL27_232.

The high-probability repeats, representative of the groups based on *Th*. *bessarabicum*:

1. TB_CL2_379nt is a common repeat, found in all species except for *Ae*. *tauschii* and somewhat similar to AC4x_CL244_376nt at the 62% identity. It is also highly similar to oligos BSCL1-1, BSCL1-2, DP4J27982, and DP4J28086 with 100% identity (see №9).

The low-probability repeats, representative of the groups based on *Th*. *bessarabicum*:

1. TB_CL148_662nt is a low-copy repeat, not found in 6x *Ae*. *crassa* and *Ae*. *tauschii* subsp. *strangulata*. It is highly similar to AC4x_CL162_661nt, and to *A*. *cristatum* ACRI_TR_CL80 satellite sequence (MG323513.1)

### Repeats’ comparative assembly analysis

The comparative analysis allowed us to find repeats unique for 4x *Ae*. *crassa*, which are absent in other studied species.

TB + ATs + AC4x_CL82_379nt and TB + ATs + AC4x_CL88_376nt are presumably *Th*. *bessarabicum*-specific, even though we get no additional information from this assembly, as the first repeat has a clear analogue in *Th*. *bessarabicum* individual assembly, and the second, in contrast with 4x *Ae*. *crassa* reads share, aligns well with AC4x known repeat. However, both of them are absent in *Ae*. *tauschii* subsp. *strangulata*. TB + ATs + AC4x_CL217_316nt and TB + ATs + AC4x_CL234_319nt are 4x *Ae*. *crassa*-specific, both found in 4x *Ae*. *crassa* single species assembly (Supplementary Table S5).

TB + ATt + AC4x_CL87_379nt and TB + ATt + AC4x_CL93_376nt are both absent in *Ae*. *tauschii* subsp. *typica* and are equivalent to the first pair of repeats described above for the previous assembly. TB + ATt + AC4x_CL204_316nt and TB + ATt + AC4x_CL220_319nt are 4x *Ae*. *crassa*-specific and are equivalent to the last pair of repeats described above for the previous assembly (Supplementary Table S6).

ATs + AC4x_CL203_316nt, ATs + AC4x_CL213_319nt, ATs + AC4x_CL247_1319nt, ATs + AC4x_CL224_178nt, and Ats + AC4x_CL240_553nt are 4x *Ae*. *crassa*-specific, and all of them have a clear analogue in 4x *Ae*. *crassa* single species assembly (Supplementary Table S7).

### *In silico* identification of X^cr^ subgenome-specific tandem repeats in 4x *Aegilops* *crassa*

Four novel tandem repeats were identified in RepeatExplorer2 output among filtered reads (Supplementary Table S3). Homology search showed that they have no similarity to previously identified tandem repeats of 4x *Ae*. *crassa*. Biased filtering (removing reads perfectly mapping on *Ae*. *tauschii* D genome) probably causes an increase of fraction of these reads of interest and makes it possible to assemble and classify assembled consensuses as tandem repeats. BLASTn search against the entire Nucleotide database showed numerous hits with other satellite and transposon sequences from other grass species for CL162 and CL244 and no hits against repeat-related sequences for CL257 and CL262.

### Assessment of copy numbers of repetitive DNA clusters using qPCR

The values of threshold cycle (Ct) were directly determined using qPCR for each repeat and for each species and relative copy number normalized against single-copy gene *VRN1*, which was calculated for each repeat (Supplementary Table S8). Decimal logarithm of relative quantity (RQ) was provided for copy number comparison (Table 3), and all repeats were conventionally (with highly probability) divided into high-copy (log_10_RQ > 4), medium-copy (2 < log_10_RQ < 4), and low-copy (log_10_RQ < 2). In 4x and 6x *Ae*. *crassa* CL3, CL18, CL209, CL170, CL8, and CL239 represented high-copy repeats; CL131 and CL2 were low-copy repeats, while others were classified as medium-copy repeats. In *Ae*. *tauschii* genome CL3, CL18, and CL170 were highly abundant, CL148, CL60, CL8, CL232, CL193, CL261, CL228, and CL27_232 - medium abundant, while others were found to be low-copy repeats. In *T*. *aestivum*, the high-copy repeats were CL3, CL18, the low-copy repeats were CL228, CL239, CL131, CL258, CL2, and CL257, while others were classified as medium-copy. In *Th*. *bessarabicum* CL244 and CL3 demonstrated high abundance, CL148, CL170, CL239, CL60, CL193, CL8, CL261, CL18, and CL27_232 were medium-copy, while others were classified as low-copy. Based on our findings, the following groups of repeats were distinguished:

- repeats that occur predominantly in genomes of *Ae*. *crassa* (4x and 6x): CL257 and CL258;
- repeats found only in genomes of wheat and *Ae*. *crassa*: CL209 and CL219;
- repeats abundant in genomes of *Ae*. *tauschii*, wheat, *Ae*. *crassa*: CL232 and CL228;
- repeats with similarly high abundance in all studied species: CL261, CL193, CL60, CL148, CL8, CL170, CL18, CL3, CL27_232;
- repeats not detected in genome of *Ae*. *tauschii*, but abundant in other species: CL244;
- repeats with high copy number in *Th*. *bessarabicum* and *Ae*. *crassa*: CL239;
- repeats with low copy number in all studied species: CL2 and CL131.

**Table 3:** Relative quantity (expressed in decimal logarithm) of the tandem repeats found in this study.

Repeat	*4x Ae. сrassa*	*6x Ae*. *сrassa*	*Ae*. *tauschii* subsp. *strangulata*	*T*. *aestivum*	*Th*. *bessarabicum*
CL3	5.32	5.83	5.85	5.03	4.72
CL8	4.18	4.10	3.60	3.93	3.12
CL18	4.76	5.06	5.00	4.36	2.71
CL60	3.60	3.74	3.60	3.37	3.42
CL131	0.84	0.73	–0.23	0.34	–0.07
CL170	4.25	4.71	4.39	3.72	3.63
CL193	3.41	3.60	3.46	3.15	3.25
CL209	4.63	4.08	–0.28	2.17	–0.07
CL219	3.74	3.40	0.69	2.76	0.59
CL228	3.17	3.12	2.95	1.72	–0.23
CL232	3.82	3.80	3.55	2.68	–0.13
CL239	3.99	4.02	0.97	0.42	3.45
CL244	3.14	3.45	–1.40	2.00	5.32
CL257	2.48	2.56	–2.52	–1.00	–1.70
CL258	3.26	3.07	0.00	–0.08	0.06
CL261	3.11	3.16	2.99	2.24	3.02
CL27_232	3.06	3.33	2.87	2.94	2.34
CL2	0.32	–0.86	–1.69	–0.42	–0.02
CL148	3.89	3.83	3.70	3.90	3.65

### Fluorescence *in situ* hybridization

FISH analysis of repeated DNA sequences currently isolated from 4x *Ae*. *crassa* (AE 742) and previously identified repeats showed that individual repeats differ in organization (dispersed vs. discrete signals), species and subgenome specificity, the number and chromosome position(s), and signal intensities (Supplementary Figures S3–S7). Five repeats were found to be homologous to already known DNA sequences: CL3 is homologous to pAs1, CL18 to FAT, CL131 to pTa-465, and CL241 to Oligo-3A1. Repeats CL2, CL8, CL60, CL148, CL170, CL193, CL209, CL219, CL228, CL232, CL239, CL244, CL257, CL258, CL261 (and its homolog CL198), and CL27_232 are considered as novel FISH probes.

Two *Ae*. *crassa* repeats, CL8 and CL60, and *Th*. *bessarabicum* repeat CL148 are distributed evenly along the entire lengths of all chromosomes irrespective of their (sub)genome affinities (Supplementary Figures S3, S7). Owing to the usefulness of these repeats for genome or chromosome identification, we excluded them from further analyses.

**Figure 1:**
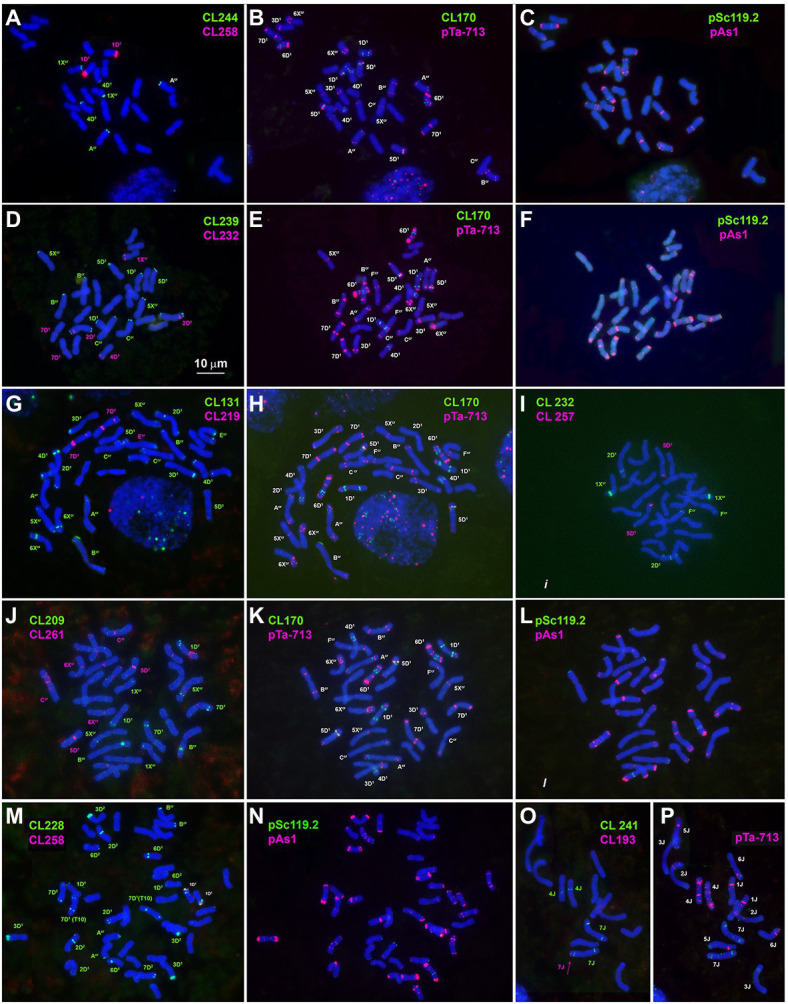
Metaphase cells of *Ae. crassa*, 4x **(A–L)** and 6x **(M,N)**, and of *Th. bessarabicum* **(O,P)** after FISH with different probe combinations; **(A–C)** the cell of 4x *Ae. crassa* AE 742 hybridized consecutively with CL244 + CL258 followed by CL170 + pTa-713 and pAs1 + pSc119.2; **(D–F)** - the cell of 4x *Ae. crassa* AE 742 hybridized consecutively with CL239 + CL232 followed by CL170 + pTa-713 and pAs1 + pSc119.2; **(G,H)** - the cell of 4x *Ae. crassa* AE 742 hybridized consecutively with CL131 + CL219 followed by CL170 + pTa-713; (I)- the cell of 4x *Ae. crassa* AE 742 hybridized with CL232 and CL257; **(J–L)** - the cell of 4x *Ae. crassa* AE 742 hybridized consecutively with CL209 + CL261 followed by CL170 + pTa-713 and pAs1 + pSc119.2; **(M,N)** – the cell of 6x *Ae. crassa* AE 131680 hybridized consecutively with CL228 + CL258 followed by pAs1 + pSc119.2; **(O,P)** – the cell of *Th. bessarabicum* PI 531711 hybridized with CL193 + CL241 followed by pTa-713. Probe combinations are given on the top of the respective images; probe color corresponds to signal color.

Other DNA probes containing already known and novel repeated sequences demonstrated discrete signals. Three of them are species-specific and produce signals on one chromosome (pair) each. Thus, CL257 and CL258 occur only in *Ae*. *crassa* (4x and 6x). Small CL257 signals appear in subterminal region of 5D^1^S arm, and CL258 signals distally on the 1D^1^S arm (Figures 1A,I,M; Supplementary Figures S3, S4). Although 6x *Ae*. *crassa* possesses two copies of the D* subgenome, only one chromosome pair belonging to D^1^ subgenome carries the signals of each of the two abovementioned probes; signals are a little more intense for CL258 than for CL257 (Figures 1A,I).

CL193 probe produces a single hybridization site on the tip of the short arm of one of the two *Th*. *bessarabicum* homologous chromosomes 7 J (Figure 1O). FISH fails to detect the CL193 on *Ae*. *crassa*, *Ae*. *tauschii*, or common wheat chromosomes.

**Figure 2:**
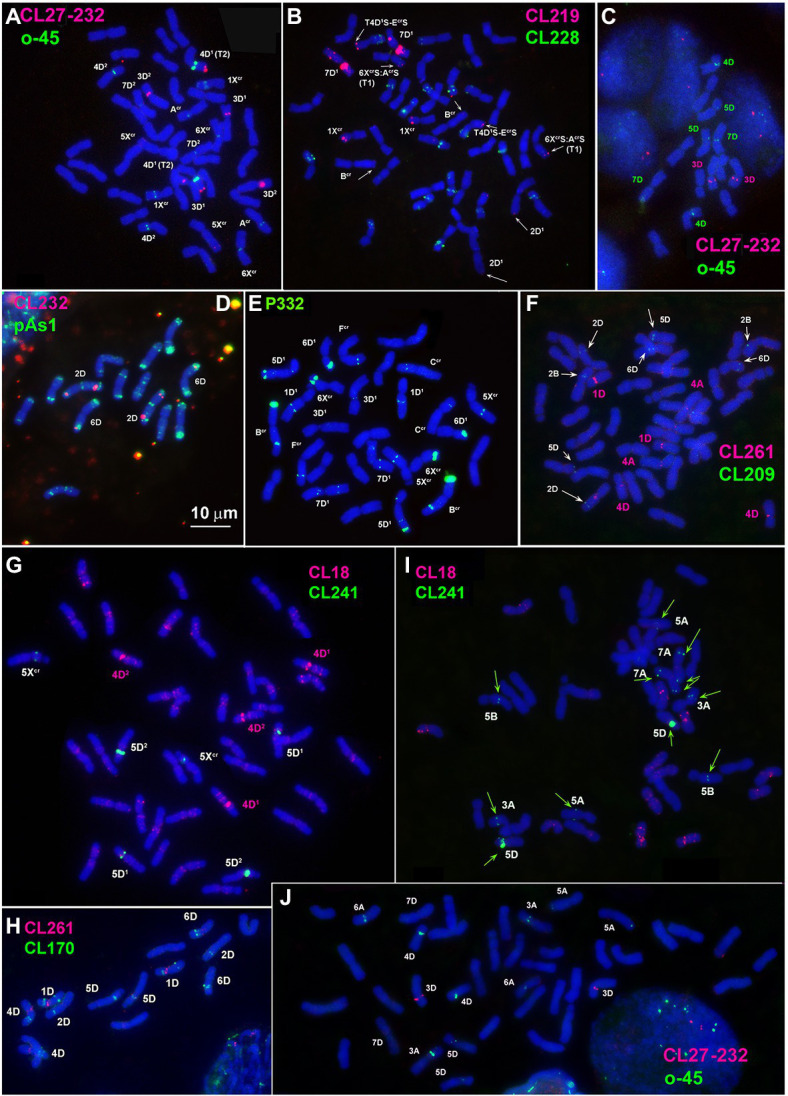
Localization of: **(A,C,J)** CL27_232 (red) + o45 (green), **(B)** CL219 (red) + CL228(green); **(D)** CL232 (red) + pAs1 (green); **(E)** P332 (green); **(F)** CL261 (red) + CL209 (green); **(G,I)** CL18 (red) + CL241 (green); **(H)** CL261 (red) + CL170 (green) on chromosomes of *Ae*. *crassa*, 4x **(E)**, 6x **(A,B,F,G)**, *Ae*. *tauschii* **(C,D,H)** and common wheat **(I,J)**.

**Figure 3:**
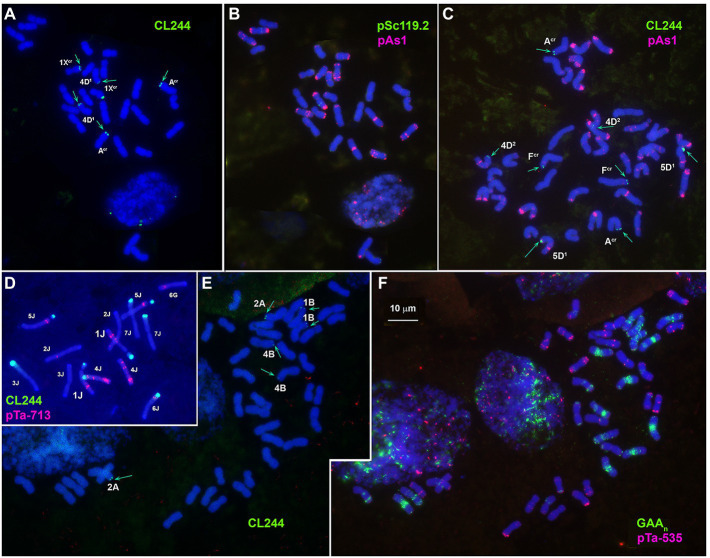
Localization of CL244 (green) repeat on *Ae*. *crassa*, 4x **(A,B)**, *Ae*. *crassa*, 6x **(C)**, *Th*. *bessarabicum* **(D)**, and common wheat **(E,F)** chromosomes. Chromosomes are identified using pAs1 (**B,C**, red), pTa-713 (**D**, red) or GAA_n_ (**F**, green) and pTa-535 (**F**, red). Chromosomes carrying CL244 signals are indicated. Scale bar, 10 mcm.

Large signals of CL27_232 probe are found in the proximal region of 3D^1^ short arms and faint signals in a distal part of 5X^cr^ long arms in both 4x and 6x cytotypes of *Ae*. *crassa* (Figures 2A; Supplementary Figures S3, S4). Hexaploid *Ae*. *crassa* possesses additional CL27_232 signals in the short arm of 3D^2^ and in the terminus of short arm of 4D^1^, which was transferred from F^cr^ short arm as a result of species-specific translocation T2 (Figure 2A; Supplementary Figure S4). Only one pair of clear CL27_232 signals is detected on chromosome 3D of *Ae*. *tauschii* (Figure 2C) and 3D of common wheat (Figure 2J).

CL244 probe displays relatively poor hybridization to *Ae*. *crassa* chromosomes. Thus, three pairs of very faint signals appear in the terminal regions of 1X^cr^L, A^cr^L, and 4D^1^S of 4x *Ae*. *crassa* (Figures 1A, 3A,B). In karyotype of 6x *Ae*. *crassa* the largest CL244 signals appear on 5D^1^S, and smaller signals - on chromosomes A^cr^L and F^cr^S. As F^cr^ was modified following species-specific translocation T2, this site is probably derived from 4D^1^S (Figure 3C). Common wheat possesses three pairs of very small CL244 signals on chromosomes 2AS, 1BS, and 4BL (Figures 3E,F), and no hybridization is detected in *Ae*. *tauschii*. On the contrary, most *Th*. *bessarabicum* chromosomes carry intense hybridization sites in either the short or long arm (Figure 3D). Signals appear on both homologous chromosomes 1JS, 5JS, and 7JS, but only on one homolog of 3JL and 6JS pairs.

The probe CL241 hybridized to the proximal part of the short arms of all group 5 chromosomes of 4x and 6x *Ae*. *crassa* (Figure 2G; Supplementary Figures S3, S4) and of *Ae*. *tauschii*. In common wheat, the CL241 sites on 5A and 5B are located in the long, but not in the short arms, while additional CL241 loci are found on 3A (long arm) and both arms of 7A (Figure 2I; Supplementary Figure S6). Chromosome 7J of *Th*. *bessarabicum* carries two CL241 sites in opposite arms (Figure 1O; Supplementary Figure S7). Additional CL241 sites are observed in the middle of 4JL, but there are no signals on group 5 chromosomes.

The CL209 signals appear mainly on the X^cr^ subgenome chromosomes of *Ae*. *crassa*. Small CL209-sites are present on chromosomes C^cr^ (middle of the short arm), 5X^cr^ (distal third of the long arm), and on a distal part of 7D^1^L arm (Figure 1J; Supplementary Figures S3, S4). Hexaploid *Ae*. *crassa* has additional large subtelomeric CL209 clusters on chromosomes 1X^cr^S and in a distal part of 6D^2^L (Figure 2F; Supplementary Figure S4). CL209 is absent in *Ae*. *tauschii* and *Th*. *bessarabicum*, but produces very weak signals on common wheat chromosomes 2BS, 2DL, and 6DS (Supplementary Figure S6).

CL219 sequence is present in abundance on *Ae*. *crassa* (4x and 6x) chromosome 7D^1^, which contains two prominent clusters located in a proximal half of the short arm in a distal third of an opposite arm (Figures 1G, 2B; Supplementary Figures S3, S4). In addition, small signals occur in a distal part of 5D^1^S and in the terminus of F^cr^S. In 6x cytotype the site from chromosome F^cr^ is transferred onto chromosome 4D^1^S following species-specific translocation T2. Hexaploid *Ae*. *crassa* also contains clear CL219 signals on three pairs of X^cr^ subgenome chromosomes: in the short arm of 1X^cr^, the long arm of 6X^cr^, and in the end of B^cr^S (Supplementary Figure S4). Common wheat possesses distinct CL219 sites in the terminal part of 2BS chromosomes and this repeat is absent in *Ae*. *tauschii* and *Th*. *bessarabicum* (Supplementary Figures S5–S7).

**Figure 4:**
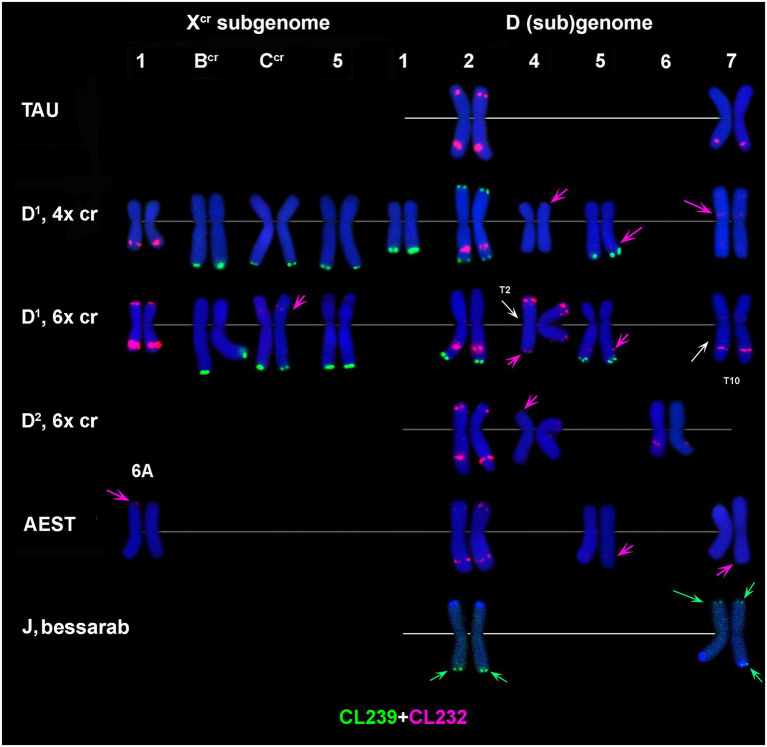
Distribution of CL239 (green) and CL232 (red) sequences on chromosomes of different cereal species: TAU – *Ae*. *tauschii* ssp. *strangulata;* D^1^, 4x cr – D^1^ subgenome of tetraploid *Ae*. *crassa*; D^1^, 6x cr, D^2^, 6x cr – D^1^ and D^2^ subgenomes of hexaploid *Ae*. *crassa;* AEST – A and D subgenomes of *T*. *aestivum*, J, bessarab – J^b^ genome of *Th*. *bessarabicum*. 1–7 – homoeologous groups. Pink arrows point to minor CL232 sites.

FISH reveals large signal of CL232 probe in a distal part of the long arm of chromosome 2D (or its derivative) in all wheat and *Aegilops* species (Figure 4, Supplementary Figures S3–S6), while a smaller site in the short arm of 2D is present only in *Ae*. *tauschii*, common wheat, and the D^2^ subgenome of *Ae*. *crassa* (6x). Prominent subtelomeric CL232 signals are detected in the long arm of 1X^cr^ in 4x (Figures 1D,I) as well as in 6x *Ae*. *crassa*, which also possesses a smaller signal terminally in the short arm (Figure 4). Hexaploid *Ae*. *crassa* has smaller signals of CL232 in a terminal part of 4D^1^S and distal quarters of 7D^1^L and 6D^2^L. Small CL232 site in 7DL appears in diploid *Ae*. *tauschii* (Figure 2D), and this sequence is absent in *Th*. *bessarabicum* genome.

CL239 is not detected in *Ae*. *tauschii* and common wheat; it is also absent in the D^2^ subgenome chromosomes of 6x *Ae*. *crassa* (Figure 4). Both *Ae*. *crassa* cytotypes contain large CL239 clusters in subterminal regions of several X^cr^ and D^1^ chromosomes (Figure 1D). Among them, five sites are common (B^cr^L, C^cr^L, 5X^cr^L, 2D^1^L, and 3D^1^), but tetraploid form contains two additional loci on 1D^1^L and 2D^1^S. Two pairwise CL239 signals and one odd signal are detected terminally on *Th*. *bessarabicum* chromosomes 2JL and 7JS + L (Supplementary Figure S7).

The largest signals of oligo-45 (o-45) probe occur in a proximal region of chromosome 4D in *Ae*. *tauschii* (Figure 2C; Supplementary Figure S5), 4x and 6x *Ae*. *crassa* (Figure 2A, Supplementary Figures S3, S4), and common wheat (Figure 2J; Supplementary Figures S6). Additional, smaller signals are found in pericentromeric regions of 5D and 7D and (*Ae*. *tauschii* and common wheat), proximal part of 3AS, subterminal region of 5AL, and pericentromeric part of 6A (common wheat, Figure 2J). *Ae*. *crassa* contain several o-45 sites located in the middle of 6D^1^S; on the chromosome A^cr^S, close to the centromere; interstitially and terminally in 1X^cr^L; in the middle of 5X^cr^L; and in pericentromere of 6X^cr^ (4x and 6x *Ae*. *crassa*) and 7D^2^ (6x *Ae*. *crassa*; Supplementary Figures S3, S4).

All studied species possess the CL261(=CL198) sequence. According to FISH, it localizes in pericentromeric regions of most chromosomes and signal intensity varies between chromosomes and between species (Figures 1J, 2F,H; Supplementary Figures S3–S7).

**Figure 5:**
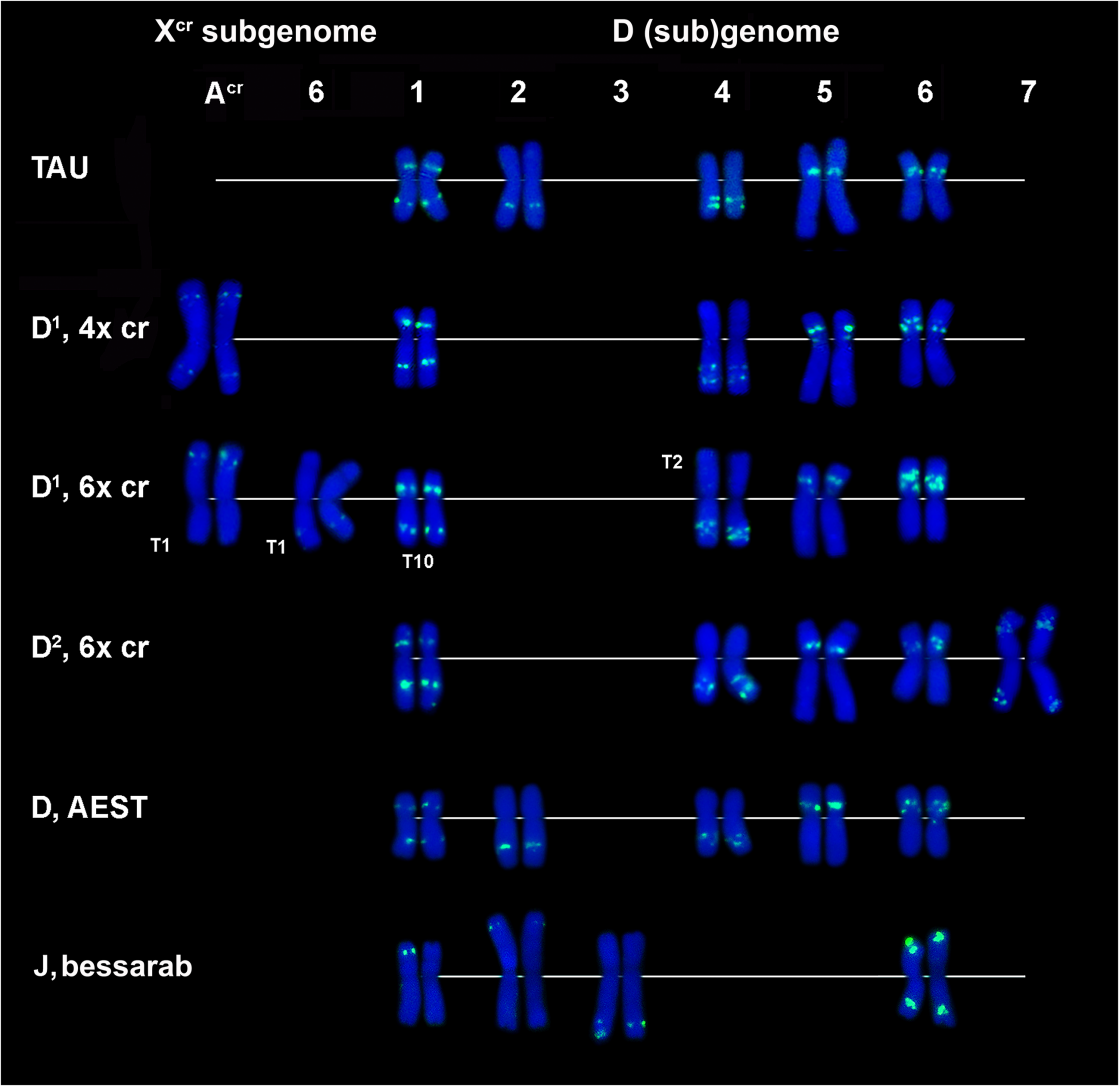
Comparison of CL170 patterns on chromosomes of different cereal species: TAU – *Ae*. *tauschii* ssp. *strangulata;* D^1^, 4x cr – D^1^ subgenome of tetraploid *Ae*. *crassa*; D^1^, 6x cr, D^2^, 6x cr – D^1^ and D^2^ subgenomes of hexaploid *Ae*. *crassa;* AEST – D subgenome of *T*. *aestivum*, J, bessarab – J^b^ genome of *Th*. *bessarabicum*. 1–7 – homoeologous groups. T1, T2, T10 – translocated chromosome of *Ae*. *crassa*. T10 causes small length reduction of chromosome region distal to CL170 site in the long arm of 1D^1^.

According to FISH, CL170 sequence is present in D (sub)genome(s) of *Triticum* and *Aegilops* species and in the J^b^ genome of *Th*. *bessarabicum* (Figure 2H; Supplementary Figures S3–S7). Distribution of CL170 sites along the D (sub)genome chromosomes is highly conserved across species (Figures 5). Two clear signals are located interstitially in the opposite arms of 1D; two signals in the middle 4DL are interrupted by a small pTa-713 site; large signal is present proximally in 5D short arm alongside a distinct pTa-713 site, and large double signals in the proximal half of 6DS (Figures 1B,E,H,K). A distinct CL170 site occurs in the middle of 2D chromosome of *Ae*. *tauschii* (Figures 2H, 5, TAU) and 2D of common wheat (Figure 5, D AEST). Two small CL170 sites are detected on opposite arms of chromosome A^cr^ of the tetraploid and in the long arms of A^cr^ and 6X^cr^ chromosomes of the hexaploid *Ae*. *crassa* cytotypes; the split of two CL170 sites between two different chromosomes is due to species-specific translocation T1. Hexaploid *Ae*. *crassa* contains CL170 sites in both, the long (subterminal) and short (double distal) arms of chromosome 7D^2^, which are absent on the orthologous chromosomes of other species.

**Figure 6:**
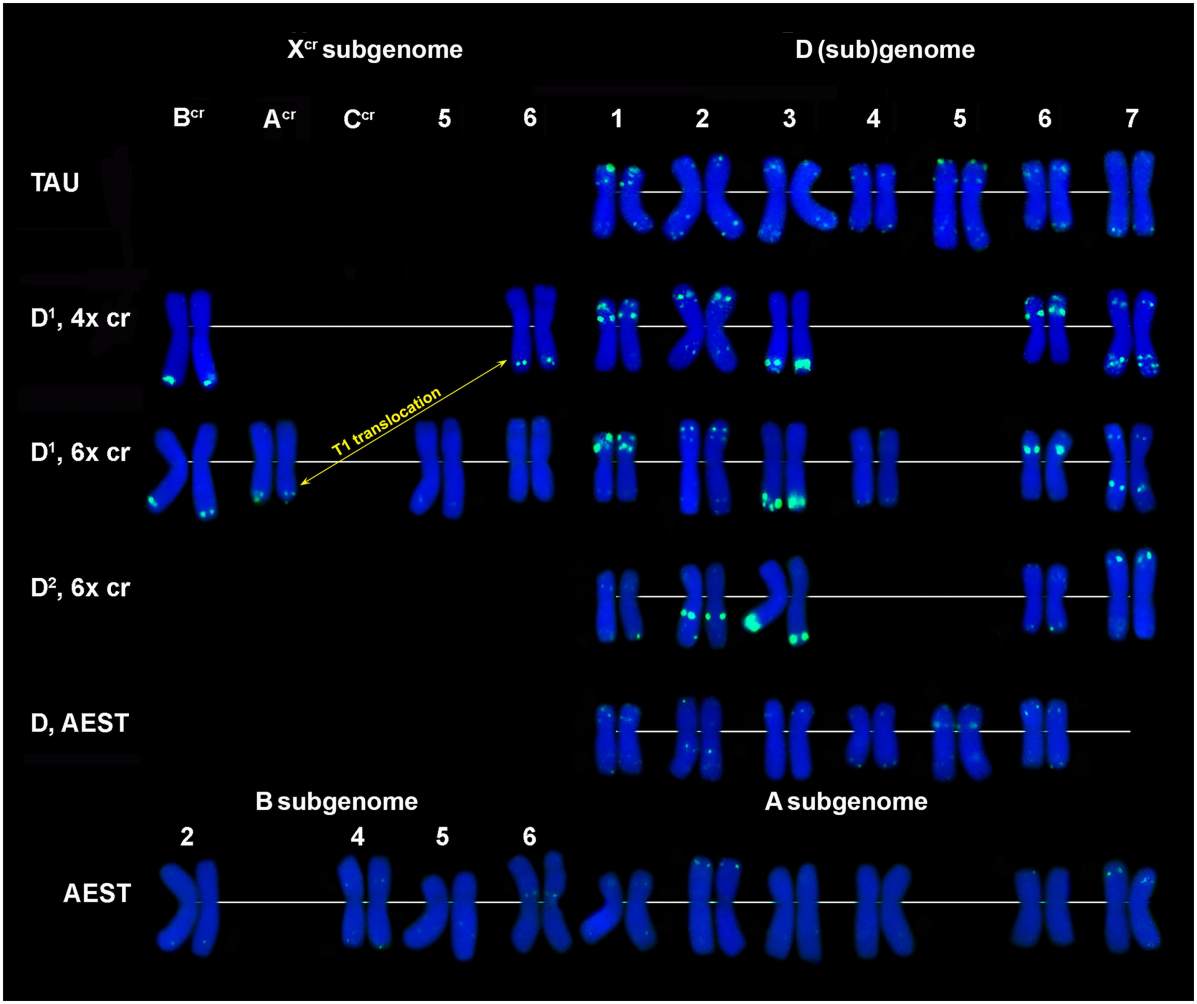
Comparison of CL228 labeling patterns on chromosomes of different cereal species: TAU – *Ae*. *tauschii* ssp. *strangulata;* D^1^, 4x cr – D^1^ subgenome of tetraploid *Ae*. *crassa*; D^1^, 6x cr, D^2^, 6x cr – D^1^ and D^2^ subgenomes of hexaploid *Ae*. *crassa;* AEST – A, B, and D subgenomes of *T*. *aestivum*. 1–7 – homoeologous groups. White arrowheads show minor CL228 sites detected on the X^cr^, A, B, and D subgenome chromosomes.

CL228 is located predominantly on the D^1^ subgenome chromosomes of 4x (Supplementary Figure S3) and 6x *Ae*. *crassa* (Figures 1M, 2B; Supplementary Figure S4). Two clear signals are detected in the terminus and in the middle part of 1D^1^ and 6D^1^ short arms; a prominent, probably double signal is observed in the terminal part of 3D^1^L, and one or a pair of small signals are present in opposite arms of 2D^1^ and 7D^1^ (Figure 6). Distinct signals are found on chromosomes B^cr^L and A^cr^L (6x *Ae*. *crassa*)/ 6X^cr^L (4x *Ae*. *crassa*), in the latter case the signal exchange is due to species-specific translocation T1.

Five pairs of the D^2^ subgenome chromosomes of 6x *Ae*. *crassa* possess CL228 sites. The largest signal occurs on 3D^2^L, similarly to 3D^1^L, and other intense sites are present in the proximal third of 2D^2^L and a distal part of 7D^2^S. Signals detected on chromosomes 1D^2^ and 6D^2^ are very weak, however their location is similar to that observed on the orthologous D^1^ subgenome chromosomes (Figure 6).

CL228 probe hybridized to all chromosomes of a diploid *Ae*. *tauschii* subsp. *strangulata*, however hybridization is much weaker compared to *Ae*. *crassa* (Figure 6; Supplementary Figure S5). Labeling patterns of the D subgenome chromosomes of common wheat are similar to that of *Ae*. *tauschii*, except for the lack of signals on 7D and less intense sites on 1D. The CL228 sequence hybridized to some A and B subgenome chromosomes of common wheat. Small but distinct signals are present on 6BS close to the centromere, in distal parts of 2AS and 7AS. Several very weak but consistent signals appear on 2B, 4B, 5B, 1A, 3A, 4A, and 6A chromosomes (Figure 6).

**Figure 7:**
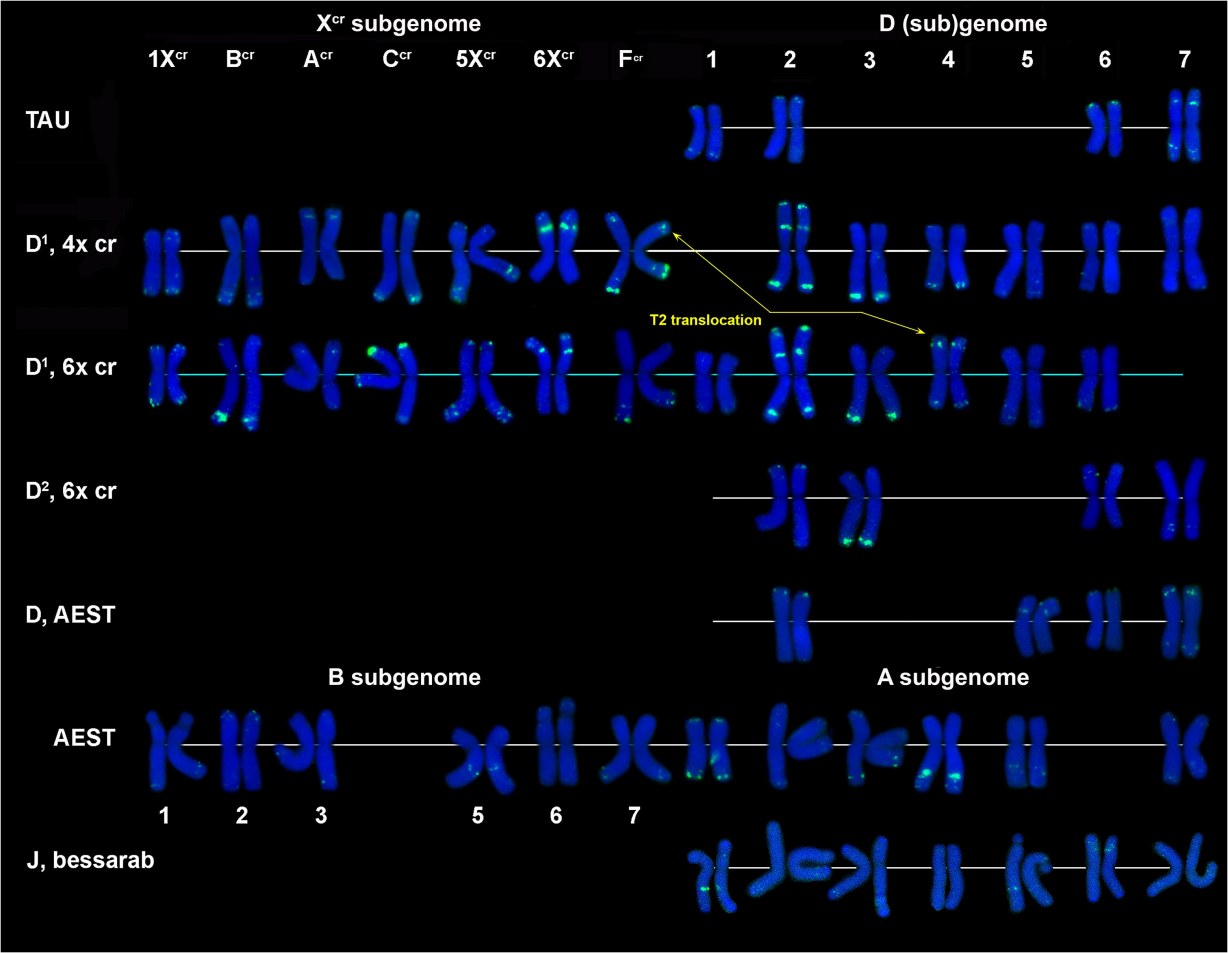
Comparison of CL131 labeling patterns on chromosomes of different cereal species: TAU – *Ae*. *tauschii* ssp. *strangulata;* D^1^, 4x cr – D^1^ subgenome of tetraploid *Ae*. *crassa*; D^1^, 6x cr, D^2^, 6x cr – D^1^ and D^2^ subgenomes of hexaploid *Ae*. *crassa;* AEST – A, B, and D subgenomes of *T*. *aestivum*; J, bessarab – J^b^ genome of *Th*. *bessarabicum*. 1–7 – homoeologous groups.

CL131 is homologous to *T*. *aestivum* clone pTa-465. CL131 hybridization patterns obtained in our study on common wheat chromosomes (Figure 7, AEST; Supplementary Figure S6) corresponds to previously reported patterns for pTa-465, thus confirming that these sequences are homologous. In common wheat and *Ae*. *tauschii* karyotypes only four out of seven D (sub)genome chromosomes carry small CL131 signals; these are 1D, 2D, 6D, and 7D in *Aegilops* and 2D, 5D, 6D, and 7D in wheat. Both *Ae*. *crassa* cytotypes carry numerous CL131 sites (Figure 1G), which have higher intensities and appear on both D^1^ and X^cr^ chromosomes (Figure 7). Most prominent sites appear on chromosome 2D^1^; subterminal signals on 3D^1^L and 3D^2^L are also large. Prominent CL131 signals are observed in the perinucleolar region of 6X^cr^ and subtelomeric parts of 7C^cr^L chromosomes; hexaploid accession also contains a clear signal on C^cr^S (Figure 7; Supplementary Figures S3, S4).

**Figure 8:**
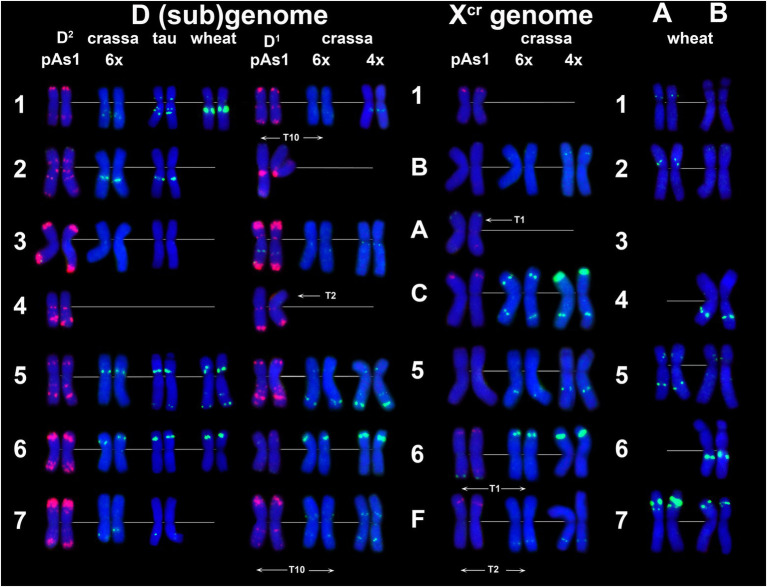
Comparison of P332 labeling patterns on chromosomes of different cereal species. D (sub)genome: D^2^ – subgenome of 6x *Ae*. *crassa*; TAU – *Ae*. *tauschii* ssp. *tauschii;* wheat – Chinese spring; D^1^ – subgenomes of tetraploid (4x) and hexaploid (6x) *Ae*. *crassa*; X^cr^ – subgenomes of tetraploid (4x) and hexaploid (6x) *Ae*. *crassa;* A and B – subgenomes of wheat (Chinese spring). 1–7 – homoeologous groups. A, B, C, F – chromosomes of *Ae*. *crassa* with unknown homoeologous group.

The P332 is homologous to pTa-k566 sequence and is similar to it in the distribution pattern on common wheat chromosomes (Figure 8). FISH detected P332 signals of variable sizes on most *Ae*. *crassa* (4x and 6x) and *Ae*. *tauschii* chromosomes (Figures 2E, 8; Supplementary Figures S3–S6), but not in *Th*. *bessarabicum*. No intraspecific variation of labeling patterns has been observed in *Ae*. *crassa*, and 4x and 6x cytotypes differ only in the size of 332 site on the chromosome C^cr^ (Figure 8). The D^1^ subgenome differs from D^2^ in labeling patterns of 2D, to a lesser extent of 5D and 7D chromosomes, and D^2^ subgenome shows high similarity with the D (sub)genomes of *Ae*. *tauschii* and common wheat.

CL18 is homologous to FAT repeat and is similar to it in chromosomal distribution. Thus, CL18 signals localize unevenly along the length of all chromosomes in all species studied. It shows more intense labeling in proximal chromosome regions (Figures 2G,I; Supplementary Figures S3–S6) being especially abundant in the chromosome(s) 4DS. The sequence CL3 is homologous to pAs1 clone and CL187 to pSc119.2. Labeling patterns of these probes are almost identical to those reported earlier for the respective sequences and are not described in this paper.

Six new repeats identified in *Ae*. *crassa*, CL8 = CL16, CL131 = CL149, CL170, CL239, CL241, and *Th*. *bessarabicum*-derived repeat CL148, were shared by *Aegilops* and *Thinopyrum* species. Two *Th*. *bessarabicum* sequences, CL16 (homologous to CL8) and CL148, are dispersed along all chromosomes; of them CL16 is more abundant in proximal, while CL148 in distal chromosome regions (Supplementary Figure S7). The probe CL198 (related to CL261) hybridizes to pericentromeric chromosome regions, while CL2, CL193, CL239, and CL244 to subterminal regions of one to several *Th*. *bessarabicum* chromosomes. A clustered pattern of CL2 is observed in terminal regions of the J^b^ genome chromosomes. Three probes, CL149 (=CL131), CL241, and CL170, hybridize to interstitial regions of *Th*. *bessarabicum* chromosomes. Signals obtained with CL131 are very faint, inconsistent, and therefore not secure for chromosome identification. Together, CL241 and CL170 can serve as reliable chromosomal markers for *Th*. *bessarabicum* (Supplementary Figure S7). For all these repeats heteromorphisms of homologous chromosomes in signal presence and/ or size is often observed.

## Discussion

Repeated nucleotide sequences are a major component of plant genome and play an important role in evolution (Salina et al., 2004b; Sharma and Raina, 2005; Dvořák, 2009; Mehrotra and Goyal, 2014; Macas et al., 2015; Liu et al., 2019). Divergence of diploid species or formation of new species *via* polyploidization are often associated with alterations in a fraction of repetitive DNA manifested in the emergence of new repeated DNA families, amplifications/eliminations of repeats, or their re-distribution between chromosomes (Zhao et al., 1998; Hemleben et al., 2007; Macas et al., 2015; Liu et al., 2019; Kuo et al., 2021; Waminal et al., 2021). Repetitive DNAs are located in structurally and functionally important chromosome regions (Heslop-Harrison et al., 2003; Shapiro and von Sternberg, 2005; Sharma and Raina, 2005; Heslop-Harrison and Schwarzacher, 2011; Mehrotra and Goyal, 2014; Garrido-Ramos, 2015) and are essential for maintenance of chromosome and genome integrity. From another side, chromosomal breaks causing chromosomal rearrangements often occur at sites enriched with repetitive DNA (Raskina et al., 2008; Molnár et al., 2011; Murat et al., 2017; Pollak et al., 2018). Owing to this, many researchers studying genome evolution and speciation in plants were focused on analysis repetitive DNA (Liu et al., 2019; Song et al., 2020; Ebrahimzadegan et al., 2021; Waminal et al., 2021). Fluorescence *in situ* hybridization is one of the most broadly exploited approaches in this field.

Development of new markers for genome studies is an important task in molecular biology and cytogenetics (Du et al., 2017; Meng et al., 2018; Said et al., 2018; Liu et al., 2019; Nikitina et al., 2020; Xi et al., 2020; Zagorski et al., 2020; Li et al., 2021; Liu and Zhang, 2021; Singh et al., 2021). Current progress in plant genome sequencing and bioinformatic analysis has opened broad perspectives for discovering new DNA sequences that can potentially be used as FISH probes, and the number of such markers rapidly increases. In our current study, as in some other publications (Liu et al., 2019; Zagorski et al., 2020; Waminal et al., 2021), we combine the benefits of molecular biology (*in vitro*), bioinformatics (*in silico*), and molecular cytogenetics (*in situ*) to get deeper insight on genome organization and karyotype evolution of *Ae*. *crassa*. The integration of different methods allowed us to identify several new repetitive DNA families, to assess their genome abundance, and map them on chromosomes by FISH. We reveal complicated genome organization of *Ae*. *crassa* and trace changes in the pattern of repetitive DNA over the course of evolution.

### Novel markers for chromosome and genome identification

The results obtained in a current study provided us more detailed information on genome and chromosome organization of *Ae*. *crassa* and the related species. We found that some repetitive sequences are widespread, whereas other sequences are restricted to particular species, subgenome(s), homoeologous group, or even a single chromosome of particular species. Thus, eight repeats isolated from *Ae*. *crassa* (CL8, CL18, CL131, CL170, CL239, CL241, CL261 = CL198, and CL244) and one *Th*. *bessarabicum* (CL148) repeats were found to be common between wheat, *Aegilops*, and *Th*. *bessarabicum*; the results of qPCR and FISH assays correlates to each other rather strongly. Importantly, at least two of these sequences, CL241 and CL170, exhibited unique labeling patterns, which differed from other FISH probes suggested for cytogenetic analysis of this grass (Du et al., 2017; Grewal et al., 2018; Chen et al., 2019; Badaeva et al., 2019b). The probes CL2 and CL244 produced large signals overlapping with the position of C-bands on *Th*. *bessarabicum* chromosomes (Mirzaghaderi et al., 2010); these sequences may be important constituents of heterochromatin blocks in this species. Taken together with CL244 and CL2 probes, the sequences CL241 and CL170 can be used as supplementary probes in FISH analysis of wheatgrass and its hybrids with common wheat.

Our analyses showed that individual *Ae*. *crassa* chromosomes differ in the number and combination of repeated families, their abundance, and physical distribution. Chromosome 5D^1^, comprising nearly all known repetitive DNA families, showed the highest diversity, while only few variants were recorded for 2D^1^ and 7X^cr^.

Five variants of repetitive DNAs hybridized exceptionally to terminal chromosome regions – CL239, CL244, CL257, CL258, and CL2 (only in *Th*. *bessarabicum*). One of the newly discovered *Ae*. *crassa* repeats, CL261, was localized in pericentromeric regions of several chromosome pairs of wheat, *Ae*. *crassa*, and *Ae*. *tauschii*. Bioinformatically, CL261 sequence was assigned to satellites (low probability) with the length of 553 nt (Supplementary Table S3). Satellite repeats are found in pericentromeric chromosome regions of many plant and animal species (Miller et al., 1998; Hudakova et al., 2001; Kishii et al., 2001; Sharma et al., 2013; Teo et al., 2013; Su et al., 2019) and CL261 could also belong to centromere-specific repeat family. CL261 showed homology to *Th*. *bessarabicum* repeat CL198, which hybridizes to pericentromeric regions of *Th*. *ponticum* chromosomes (Nikitina et al., 2020) and centromere-specific repeat CentT550 identified in common wheat (Su et al., 2019).

The following sequences occurred in subterminal and/or interstitial positions of several *Ae*. *crassa* chromosomes: CL131, CL170, CL209, CL219, CL228, CL232, CL131, CL241, and CL27_232. Particular sites, for example, proximal parts of 4D^1^S, 5D^1^S, and 7D^1^S, were especially enriched with satellite DNA and may contain several different variants of repeats. Thus, large signals of GAA_n_ (Abdolmalaki et al., 2019; Badaeva et al., 2021), CL170, CL241, and CL18 overlapping with faint signals of pTa-713 and pTa-k566 probes are found in the short arm of 5D^1^. C-banding analysis (Badaeva et al., 1998, 2002) revealed two distinct heterochromatin blocks in this chromosomal region. Another satellite-rich cluster was detected in the proximal part of chromosome 7D^1^S, which possessed an abundant quantity of CL219 and pTa-713 repeats and much lesser amounts of CL232, CL18, and pTa-k566 sequences. As in a previous case, a large Giemsa band was observed in the respective chromosome region (Badaeva et al., 1998, 2002).

Repeats CL27_232 and CL241 found in *Triticum-Aegilops* species occupied a similar position on the orthologous chromosomes. CL27_232 clusters appeared in the short arm of 3D of common wheat, *Ae*. *crassa*, and *Ae*. *tauschii*. Although few additional minor signals have been detected on chromosomes 4D^1^ (T2) and 5X^cr^ of 6x *Ae*. *crassa*, CL27_232 repeat can be used as a marker of the short arm of 3D chromosome. Such chromosome-specific markers based on single-copy genes (Danilova et al., 2014) or pooled oligo-probes (Li et al., 2021) have been developed for wheat and found broad application in phylogenetic studies of the Triticeae (Danilova et al., 2017). Repeat CL241 was found in the short arms of all homoeologous group 5 chromosomes of *Ae*. *crassa* and *Ae*. *tauschii*. This syntheny however was disturbed in wheat and *Thinopyrum* species, which possessed clear CL241 sites on A, B, and J^b^ (sub)genome chromosomes, but in different positions.

Two sequences, CL170 and CL228, occurred mainly in the D^1^ subgenome. Hybridization pattern of CL170 probe was highly conserved across the D (sub)genomes of wheat and *Aegilops* species (Figure 5), whereas more intense hybridization sites of CL228 on *Ae*. *crassa* chromosomes compared to *Ae*. *tauschii* or common wheat (Figure 6) suggested sequence amplification following speciation of this amphiploid. Interestingly, labeling patterns of CL170, CL244 probes on *Ae*. *tauschii* subsp. *strangulata* and D subgenome chromosomes of common wheat differed from that in the D^1^ and D^2^ chromosomes, suggesting that these differences may exist in the genome of diploid progenitor. This assumption is legitimate, as many molecular (Luo et al., 2007; Wang et al., 2013; Li et al., 2018; Singh et al., 2019; Gaurav et al., 2021) and cytogenetic (Majka et al., 2017; Zhao et al., 2018; Badaeva et al., 2019a; Ebrahimzadegan et al., 2021) data supported significant genome divergence between two *Ae*. *tauschii* subspecies.

Two repeats were more abundant in the X^cr^ subgenome, CL131 homologous to pTa-465 and P332 homologous to pTa-k566 (Komuro et al., 2013), but occurred also in D^1^ and D^2^ subgenomes (Figures 7; 8, Supplementary Figures S3, S4). Both probes hybridized to subtelomeric and interstitial chromosome regions and showed different labeling patterns between D genomes of *Ae*. *tauschii* subsp. *strangulata* and *tauschii* vs. D^1^ and D^2^ subgenome chromosomes of *Ae*. *crassa*. The CL131 is, according to our current results, more abundant in the X^cr^ rather than D^1^ subgenome of *Ae*. *crassa* (Figure 7). Thus, three prominent CL131 clusters were detected on 2D^1^ and another one, overlapping with CL228, on 3D^1^L. Such prominent CL131 (pTa-465) clusters were not found in wheat (Komuro et al., 2013) or in subsp. *strangulata* (accession K-112), but smaller pTa-465 sites in similar positions were recorded in several *Ae*. *tauschii* accessions by Majka et al. (2017). Unfortunately, these authors did not provide full taxonomic description on the material they used.

Most CL131 sites on X^cr^ chromosomes were small and only 6X^cr^ possessed large signals comparable in size with signals on chromosome 4A of wheat. We found differences between 4x and 6x accessions in the size of CL131 sites on chromosomes C^cr^S and F^cr^L, which are probably not related to the formation of hexaploid form, whereas modifications of labeling patterns of F^cr^S and 4D^1^S chromosomes were likely caused by species-specific translocation T2. No traces of CL131 repeat were recorded on *Th*. *bessarabicum* chromosomes by FISH, which corresponds to qPCR results.

According to bioinformatics and qPCR the novel CL239 repeat is absent in the *Ae*. *tauschii* genomes. Indeed, FISH detected CL239 sites on most X^cr^ chromosomes, but only on two D^1^ subgenome chromosomes. In addition to *Ae*. *crassa*, CL239 was discovered in *Th*. *bessarabicum*, but it was absent in the D (sub)genomes of wheat and *Ae*. *tauschii*, and the D^2^ subgenome of 6x *Ae*. *crassa*, in agreement with results obtained by other methods. Based on these observations we suggest that CL239 sequence was probably contributed to *Ae*. *crassa* by the putative progenitor of X^cr^ subgenome, and it spread to the D^1^ subgenome chromosomes following species evolution. Alternatively, this sequence could present in a putative progenitor of the D^1^ subgenome, but after formation of primary allopolyploid was amplified in polyploid descendant, but eliminated from diploid ancestor. Amplification of CL131 and CL239 repeats in *Ae*. *crassa* can be suggested based on the lack of this sequence in *Ae*. *tauschii* genome. Comparison with 6x *Ae*. *crassa* however showed that this is true only for CL239, because most D^2^ subgenome chromosomes had a CL131 hybridization pattern similar with the D^1^ chromosomes.

Although CL232 repeat hybridized only on a few *Ae*. *crassa* chromosomes, it helped us to shed some light on the structure of chromosome 2D^1^, the origin of which is still highly speculative. Labeling patterns of all repeats used in our current and in all previous studies (Badaeva et al., 1998, 2002, 2021; Abdolmalaki et al., 2019) showed that most drastic changes occurred in this *Ae*. *crassa* chromosome as compared to *Ae*. *tauschii*. Chromosome 2D^1^ was assigned to the D subgenome because i) it showed distinct hybridization with the D genome-specific probe pAs1; and ii) labeling pattern of CL232 and CL239 probes on the long arm of 2D^1^ were almost identical to the long arm of 2D orthologs of *Ae*. *tauschii*, the D^2^ subgenome of 6x *Ae*. *crassa*, and D subgenome of common wheat. At the same time, 2D^1^ lacks hybridization sites of CL170, CL228, and pTa-k566, and acquires three CL131 clusters, which point to significant structural rearrangement of *Ae*. *crassa* chromosome 2D^1^.

Comparison of the results of the preliminary estimation of the repeats abundance using qPCR and the localization of the identified repeats to the chromosomes of the studied species generally showed the collinearity of the results obtained by these methods. Thus, CL8, CL18, CL60, and CL148 demonstrated a high copy number and noticeable dispersed signals on the chromosomes in all studied species. CL3, CL261, CL27_232, and CL170 also showed high copy number in all studied species according to qPCR results, and discrete pattern of hybridization on chromosomes. It can also be noted that the species specificity revealed by the qPCR results was also observed when analyzing the FISH results: CL257 and CL258 are found only in *Ae*. *crassa*, CL209 and CL219 in *Ae*. *crassa* and *T*. *aestivum*, CL228 and CL232 in *Ae*. *crassa*, *Ae*. *tauschii* and less in wheat, absence of CL239 in wheat and *Ae*. *tauschii*, absence of CL244 in *Ae*. *tauschii*, and high abundance and bright signals on the *Th*. *bessarabicum* chromosomes.

However, we also revealed differences between qPCR data and localization of repeats on chromosomes using FISH for CL193, CL2, and CL131. Although CL193 showed a high copy number in all species in the qPCR experiment, it hybridized to one pair of *Th*. *bessarabicum* chromosomes only. CL193 is homologous to the dispersed-clustered FAT and P631 repeats, as well as to the microsatellite-related FISH-positive repetitive sequence pTa-451 (Supplementary Table S9). We can assume that the CL193 repeat is quite strongly dispersed throughout the chromosomes without cluster localization, so that FISH is not able to identify its presence. On the other hand, qPCR in this case could give false positive results due to primer annealing and non-specific amplification on other repeating elements. CL2, homologous to terminal repeats of various Triticeae species (Supplementary Table S9), in our experiments, was absent in the studied species by qPCR, but showed bright terminal signals on the *Th*. *bessarabicum* chromosomes. Similarly, CL131 also showed extremely low abundance in qPCR, but showed clear local signals on the chromosomes of all species studied. In these cases, qPCR showed false-negative results, which can be explained by the low efficiency of the selected primers or difficult amplification regions for the polymerase. Nevertheless, despite individual cases, in general, the qPCR method has demonstrated its suitability for preliminary screening of novel DNA repeats for their application as chromosomal markers.

### Evolutionary changes of repetitive DNA families in *Aegilops* *crassa* genome

The five repeats we identified, CL2, CL239, CL244, CL257, and CL258, are distinguished by their conservative subtelomeric localization. Sequence analysis showed that CL2, which was found in *Th*. *bessarabicum* genome, is homologous to terminal/subterminal repeats found in *Th*. *bessarabicum*, *Leymus racemosus*, *Dasypyrum villosum*, and *Secale cereale* (Wilkes et al., 1995; Francki et al., 1997; Kishii et al., 1999; Pace et al., 2011; Du et al., 2017; Chen et al., 2019), as well as to CL244; the CL244 repeat itself showed homology to terminal repeats of *Th*. *bessarabicum*, *Ae*. *speltoides* (Spelt52.1), and *S*. *cereale* (including pSc200; Appels et al., 1981; Vershinin et al., 1995; Salina et al., 2004a; Du et al., 2017; Chen et al., 2019), whereas CL239 is homologous to Spelt1-like repeat Tri-MS-6 (EF469549.1; Supplementary Table S9). Subtelomeric repeats are localized in terminal heterochromatic blocks and their copy number may vary between accessions and between species and can change during evolution. Moreover, even homologous FISH probes may give different signals (or no signals at all) in the same species. For example, oligo-probes DP4J20764 and DP4J30938, which are homologous to CL2, gave signals on chromosomes of *Th*. *bessarabicum* and wheat, while the DP4J31304 was found only in chromosomes of *Th*. *bessarabicum* (Du et al., 2017). CL2 hybridized only to *Th*. *bessarabicum* chromosomes, while CL244 and CL239 showed hybridization to chromosomes of *Th*. *bessarabicum*, as well as the X^cr^ and D^1^ subgenome chromosomes of *Ae*. *crassa*, but absent in *Ae*. *tauschii*. Judging from the wide range of genomes in which the terminal repeats CL2, CL239, and CL244 (V, R, J, S) occur, we can propose their antiquity and even possible common origin of CL2 and CL244. The CL2, CL239, and CL244 repeats can probably be amplified during *Th*. *bessarabicum* speciation, but CL2 and CL239 were totally eliminated from *Aegilops* and *Triticum*, while CL244 was retained in the S, R, X^cr^ genomes and the putative ancestor of wheat B subgenome. Signals on D and A subgenome chromosomes of common wheat may appear due to the transfer of CL244 repeat from B to the A subgenome chromosomes, while CL239 and CL244 may be transferred from X^cr^ to the D^1^ subgenome chromosomes of *Ae*. *crassa* after polyploidization, as a result of coevolution of subgenomes, as was supposedly for terminal repeats Spelt1 and Spelt52 (Zoshchuk et al., 2007; Raskina et al., 2011).

Subtelomeric repeats play an important role in recognition of homologous chromosomes during meiosis (Corredor et al., 2007; Aguilar and Prieto, 2020). We found CL257 and CL258 repeats only in the D^1^ subgenome of the *Ae*. *crassa* (4x and 6x) on chromosomes belonging to genetic groups 1 and 5, respectively, which may indirectly indicate their role in the recognition of these chromosomes during meiosis and promote their differentiation from homoeologues. In turn, CL244 forms signals of varying intensity in termini of several X^cr^ and D^1^ chromosomes, which may also indirectly indicate their putative involvement in chromosome recognition in meiosis.

CL244, which was filtered bioinformatically as the putative X^cr^ subgenome-specific repeat, may also exhibit partial sequence elimination in *Ae*. *crassa*. Only a few faint FISH signals were observed on *Ae*. *crassa* and common wheat chromosomes, and it was totally absent in *Ae*. *tauschii*, which agrees with qPCR results. Despite some polymorphisms in CL244 location, the signals were always found in subtelomeric regions of wheat and *Aegilops* chromosomes, and most chromosomes carrying CL244 sites belong to subgenomes other than D/ D^1^. According to both FISH and qPCR analyses, CL244 is highly abundant in *Th*. *bessarabicum*, which presumed that this repeat was inherited from an ancient grass ancestor and remained mainly in J^b^ and X^cr^ (sub)genomes.

Only two repetitive DNA families analyzed in our study, CL257 and CL258, were found to be species-specific. Each of them was mapped to a single *Ae*. *crassa* chromosome, 5D^1^ and 1D^1^, respectively, and in both cases hybridization signals had terminal location. This is not surprising because many pieces of experimental evidence prove that species-specific repeats are often accumulated in subtelomeric regions of plant chromosome, which comprise rapidly evolving families of satellite repeats (Anamthawat-Jonsson and Heslop-Harrison, 1993; Salina et al., 2004b; Lim et al., 2005; Sharma and Raina, 2005; González-García et al., 2006). Bioinformatically, CL257 and CL258 were classified as “true satellites,” which occur in genome of *Ae*. *crassa* with the same frequency of 0.019% (Supplementary Table S3). Real-time qPCR confirmed their presence in both 4x and 6x *Ae*. *crassa* and absence in other species (Supplementary Table S8). None of the methods we used here revealed these repeats in other species, and no hits have been found in NCBI database. Based on these observations we assume that these two repeats emerged *de novo* in the D^1^ subgenome at the stage of formation of primary amphiploids or during subsequent evolution of 4x *Ae*. *crassa*. Probably owing to this novelty, bioinformatics attributed the CL257 repeat to the X^cr^ subgenome, although physically it localizes on the D^1^ chromosome that emphasize the necessity of complex approaches in repeatome studies. The emergence of five novel *Ae*. *crassa*-specific repetitive DNA sequences was earlier documented by Dubkovsky and Dvořák (1995), however, the relationships of these repeats with CL257 and CL258 remain not known. No differences in CL257 and CL258 signal intensities have been observed between 4x and 6x *Ae*. *crassa*, suggesting that hexaploidization did not cause significant changes in their content.

Formation of 4x *Ae*. *crassa* was associated with massive amplification of certain satellite repeats that may already pre-exist in the genome of the putative progenitor species. This mechanism was probably responsible for the emergence of two huge CL219 clusters on 7D^1^ in both 4x and 6x *Ae*. *crassa*. According to FISH, this sequence is lacking on the orthologous chromosomes of common wheat, *Ae*. *tauschii*, and on 7D^2^ of *Ae*. *crassa*. On the other side, CL219 was absent in *Th*. *bessarabicum* and *Ae*. *tauschii*, but occurred on chromosomes 2BS of common wheat and 1X^cr^, 6X^cr^, and 7X^cr^ of *Ae*. *crassa*. All these chromosomes belong to subgenomes other than D, and all sites were only found in subtelomeric regions. Based in these observations, we assumed that CL219 satellite was present in minor quantities in the putative X^cr^ subgenome progenitor. It could probably be transferred to the proximal region of 7D, which was highly enriched with other satellite sequences (e.g., pTa-713, pTa-k566, CL18, and CL232) *via* the mechanism of ectopic pairing (Schubert and Lysak, 2011; Waminal et al., 2021) soon after formation of primary *Ae*. *crassa* amphiploid. Genomic shock might cause massive amplification and spread of the repeat to other chromosomal sites, leading to the emergence of prominent CL219 clusters in proximal (7D^1^S) and distal (7D^1^L) chromosome regions.

We identified two repeats, CL261 and CL198, localized in the pericentromeric region, which are found to be homologous to the centromeric repeats CentT550 and 17–202 (Supplementary Table S9). Centromeres play an important role in the precise segregation of sister chromatids in mitosis and meiosis, mediated by the centromere-specific histone protein CENH3. Localization of this protein coincides with the centromeric arrangement of CentT550 repeats, which is characteristic mainly for the D subgenome of wheat (and *Ae*. *tauschii*), and CentT566, which is characteristic mainly for the B subgenome of wheat (and *Ae*. *speltoides*) and, probably, served as a source of these repeats in other subgenomes of bread wheat after polyploidization (Mach, 2019). Repeat 17–202 was found in the genome of *Th*. *pontium* and is localized to chromosomes of both wheat and wheatgrass (Nikitina et al., 2020). The CL261 repeat is localized mainly on the D^1^ chromosomes of 4x and 6x *Ae*. *crassa* and on the D subgenome chromosomes of common wheat. Its analogue, CL198, is localized on five pairs of *Th*. *bessarabicum* chromosomes. Thus, most likely we identified a new centromeric repeat that has a common origin with CentT550 and passed to both the J and D (sub)genomes of wheatgrass and *Aegilops* during evolution. Considering the importance of this repeat in ensuring proper meiosis, we can assume that the simultaneous presence of CentT550-like repeats in wheat and wheatgrass chromosomes of intergeneric hybrids may explain predominant introgressions of the J genome chromatin of *Thinopyrum* to the D subgenome of wheat.

We also identified a few chromosome-or group-specific repeats. Thus, CL241, which is homologous to Oligo-3A1 (Lang et al., 2018; Lang et al., 2019b) and Oligo-44 (Tang et al., 2018; Supplementary Table S9), was mapped to the same wheat or *Ae*. *tauschii* chromosomal regions as was described in literature. Their signals are predominantly found on group 5 chromosomes, either in the short [(sub)genomes D, S^l^, S^s^] or long [(sub)genomes S, B, A] arms. In *Th*. *bessarabicum*, we detected signals on two chromosome pairs (with one and two hybridization sites), while Lang et al. (2019b) identified two and four chromosomes carrying a single Oligo-3A1 site each in *Th*. *ponticum* and *Th*. *intermedium*, respectively. We did not find this repeat in the X^cr^ subgenome, Lang et al. (2019b) did not detect it in barley and *Dasypyrum breviaristatum*. Thus, we can assume that the X^cr^ subgenome donor diverged from a common ancestor after barley and *D*. *breviaristatum*, but prior to active amplification of this repeat in *Triticum*/*Aegilops*/*Thinopyrum*/*D*. *villosum*.

We can reach a similar conclusion considering that the CL170 repeat is absent in X^cr^ genome, but is abundant in the D (sub)genome of wheat and *Ae*. *tauschii*, *Ae*. *crassa*, and *Th*. *bessarabicum*. The homologous sequences were detected on chromosomes of *Th*. *ponticum*, *Th*. *bessarabicum*, *Th*. *intermedium*, *A*. *cristatum*, but they were absent in *D*. *villosum* and *Pseudoroegneria spicata* chromosomes (Supplementary Table S9). Probably, the putative X^cr^ genome progenitor diverged from a common ancestor after separation of the V and St genomes, and prior to the start of massive amplification of the CL170-like repeat in *Triticum*/*Aegilops*/*Thinopyrum*/*A*. *cristatum*.

Results obtained in our study showed that evolution of *Ae*. *crassa* was associated not only with amplification, but also with elimination of repetitive DNA sequences, as was described for other plant species (Dvořák and Zhang, 1992; Salina et al., 2004b; Adams and Wendel, 2005; Feldman and Levy, 2005; Kumar et al., 2010). For example, bioinformatic analysis failed to detect sequence AC4x_CL193_504nt in *Th*. *bessarabicum* J^b^ genome; qPCR showed its high abundance in genomes of all analyzed species, while FISH detected a single CL193 signal on one of the two homologs 7 J, and no signals were found in *Ae*. *crassa*. Probably, this sequence was present in the genome of the putative progenitor(s) of *Ae*. *crassa*, but it was eliminated during evolution of *Aegilops* species. *Ae*. *crassa*, being the most ancient polyploid species in the genus *Aegilops*, may retain a tracing amount of this repeat, which cannot be detected at chromosomal level by FISH.

Thus, if the full-length genome assemblies are unavailable, identification of new repetitive DNA sequences in particular species based on results of low-coverage NGS sequencing makes it possible to broaden the pool of data and get closer to their analysis at the level of “omics” technologies with lesser time and financial expenses.

## Data availability statement

The original contributions presented in the study are publicly available. This data can be found here: NCBI, ON872662–ON872692.

## Author contributions

EB, GK, and MD conceived and designed the experiments, analyzed data, interpreted results, wrote and edited the manuscript. EB, VS, TK, and AY designed and conducted the cytogenetic experiments and analyzed chromosome images. NC and MB provided and botanically verified plant material for analysis. SS designed and synthesized oligo-probes for FISH analyses. EN, DU, and AE performed bioinformatics analysis. AK performed and analyzed qPCR experiments. EB, OR, and PK wrote the first manuscript version. All authors contributed to the article and approved the submitted version.

## Funding

This research was funded by the Russian Science Foundation, grant number 21-16-00123.

## Conflict of interest

The authors declare that the research was conducted in the absence of any commercial or financial relationships that could be construed as a potential conflict of interest.

## Publisher’s note

All claims expressed in this article are solely those of the authors and do not necessarily represent those of their affiliated organizations, or those of the publisher, the editors and the reviewers. Any product that may be evaluated in this article, or claim that may be made by its manufacturer, is not guaranteed or endorsed by the publisher.
