# Erratum: *Aegilops crassa* Boiss. repeatome characterized using low-coverage NGS as a source of new FISH markers: application in phylogenetic studies of the Triticeae

**DOI:** 10.3389/fpls.2023.1207880

**Published:** 2023-07-13

**Authors:** Pavel Yu. Kroupin, Ekaterina D. Badaeva, Victoria M. Sokolova, Nadezhda N. Chikida, Maria Kh. Belousova, Sergei A. Surzhikov, Ekaterina A. Nikitina, Alina A. Kocheshkova, Daniil S. Ulyanov, Aleksey S. Ermolaev, Thi Mai Luong Khuat, Olga V. Razumova, Anna I. Yurkina, Gennady I. Karlov, Mikhail G. Divashuk

**Affiliations:** ^1^All-Russia Research Institute of Agricultural Biotechnology, Kurchatov Genomics Centre – ARRIAB, Moscow, Russia; ^2^N.I.Vavilov Institute of General Genetics, Russian Academy of Sciences, Moscow, Russia; ^3^Engelhardt Institute of Molecular Biology, Russian Academy of Sciences, Moscow, Russia; ^4^All-Russian Institute of Plant Genetic Resources (VIR), Department of Wheat Genetic Resources, St. Petersburg, Russia; ^5^Agricultural Genetics Institute, Department of Molecular Biology, Hanoi, Vietnam

**Keywords:** *Aegilops*, shallow whole-genome sequencing, fluorescence *in situ* hybridization, chromosomes, satellite repeats, repeatome

In the published article, there was an error in the legend for **Figure 1**, page ten. The accession number of *Th*. *bessarabicum* used in the study was indicated erroneously.

**Figure 1:**
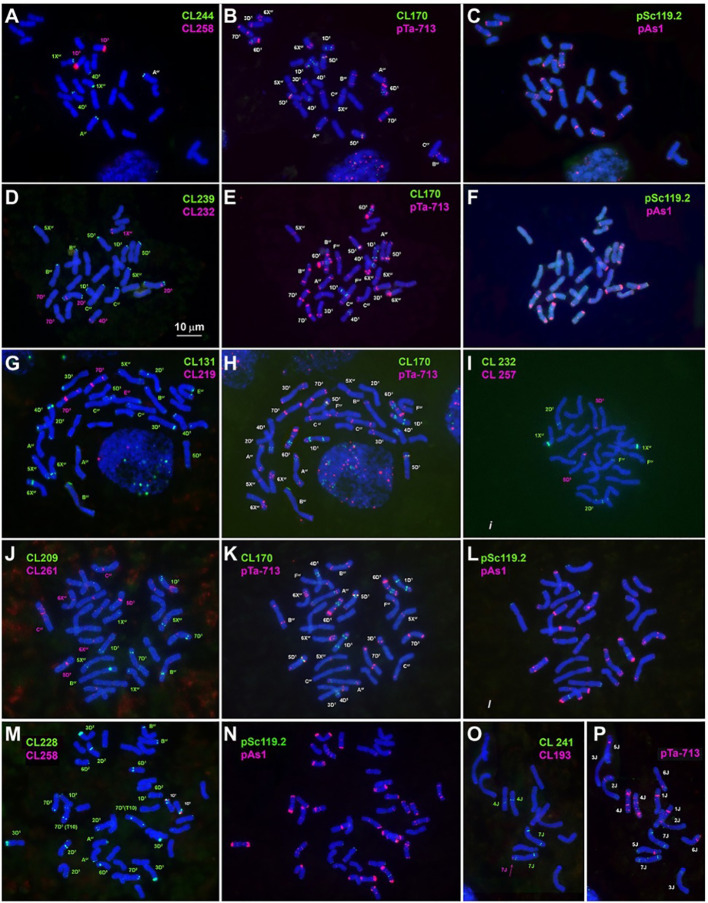
Metaphase cells of *Ae. crassa*, 4x **(A–L)** and 6x **(M, N)**, and of *Th. bessarabicum* **(O, P)** after FISH with different probe combinations; **(A–C)** the cell of 4x *Ae. crassa *AE 742 hybridized consecutively with CL244 + CL258 followed by CL170 + pTa-713 and pAs1 + pSc119.2; **(D–F)** - the cell of 4x *Ae. crassa* AE 742 hybridized consecutively with CL239 + CL232 followed by CL170 + pTa-713 and pAs1 + pSc119.2; **(G, H)** - the cell of 4x *Ae. crassa *AE 742 hybridized consecutively with CL131 + CL219 followed by CL170 + pTa-713; **(I)** the cell of 4x *Ae. crassa* AE 742 hybridized with CL232 and CL257; **(J–L)** - the cell of 4x *Ae. crassa* AE 742 hybridized consecutively with CL209 + CL261 followed by CL170 + pTa-713 and pAs1 + pSc119.2; **(M, N)** – the cell of 6x *Ae. crassa* AE 131680 hybridized consecutively with CL228 + CL258 followed by pAs1 + pSc119.2; **(O, P)** – the cell of *Th. bessarabicum* PI 531711 hybridized with CL193 + CL241 followed by pTa-713. Probe combinations are given on the top of the respective images; probe color corresponds to signal color.

The legend previously stated:

**“** **Figure 1**

Metaphase cells of *Ae*. *crassa*, 4x **(A–L)** and 6x **(M,N)**, and of *Th*. *bessarabicum* **(O,P)** after FISH with different probe combinations; **(A–C)** the cell of 4x *Ae*. *crassa* AE 742 hybridized consecutively with CL244 + CL258 followed by CL170 + pTa-713 and pAs1 + pSc119.2; **(D–F)** - the cell of 4x *Ae*. *crassa* AE 742 hybridized consecutively with CL239 + CL232 followed by CL170 + pTa-713 and pAs1 + pSc119.2; **(G,H)** - the cell of 4x *Ae*. *crassa* AE 742 hybridized consecutively with CL131 + CL219 followed by CL170 + pTa-713; **(I)**- the cell of 4x *Ae*. *crassa* AE 742 hybridized with CL232 and CL257; **(J–L)** - the cell of 4x *Ae*. *crassa* AE 742 hybridized consecutively with CL209 + CL261 followed by CL170 + pTa-713 and pAs1 + pSc119.2; **(M,N)** – the cell of 6x *Ae*. *crassa* AE 131680 hybridized consecutively with CL228 + CL258 followed by pAs1 + pSc119.2; **(O,P)** – the cell of *Th*. *bessarabicum* PI 201890 hybridized with CL193 + CL241 followed by pTa-713. Probe combinations are given on the top of the respective images; probe color corresponds to signal color.”

The corrected legend appears below:

**“** **Figure 1**

Metaphase cells of *Ae*. *crassa*, 4x **(A–L)** and 6x **(M,N)**, and of *Th*. *bessarabicum* **(O,P)** after FISH with different probe combinations; **(A–C)** the cell of 4x *Ae*. *crassa* AE 742 hybridized consecutively with CL244 + CL258 followed by CL170 + pTa-713 and pAs1 + pSc119.2; **(D–F)** - the cell of 4x *Ae*. *crassa* AE 742 hybridized consecutively with CL239 + CL232 followed by CL170 + pTa-713 and pAs1 + pSc119.2; **(G,H)** - the cell of 4x *Ae*. *crassa* AE 742 hybridized consecutively with CL131 + CL219 followed by CL170 + pTa-713; **(I)**- the cell of 4x *Ae*. *crassa* AE 742 hybridized with CL232 and CL257; **(J–L)** - the cell of 4x *Ae*. *crassa* AE 742 hybridized consecutively with CL209 + CL261 followed by CL170 + pTa-713 and pAs1 + pSc119.2; **(M,N)** – the cell of 6x *Ae*. *crassa* AE 131680 hybridized consecutively with CL228 + CL258 followed by pAs1 + pSc119.2; **(O,P)** – the cell of *Th*. *bessarabicum* PI 531711 hybridized with CL193 + CL241 followed by pTa-713. Probe combinations are given on the top of the respective images; probe color corresponds to signal color.”

In the published article, there was an error in **Table 1**, page four. The genomes D^1^ and D^2^ were mistyped, the accession number of *Th*. *bessarabicum* used in the study was indicated erroneously, and the source of *Th. bessarabicum* accessions was incorrect.

**Table 1:** Plant material.

Species	2n	Genome formula	Accessions #	Used in:	Source
*Ae. crassa*	28	D^1^D^1^X^cr^X^cr^	AE 742	Sequencing, qPCR, FISH	IPK, Gatersleben, Germany
			AE 1649	FISH	
			K-2485	FISH	VIR, St.-Petersburg, Russia
*Ae. crassa*	42	D^1^D^1^X^cr^X^cr^D^2^D^2^	IG 131680	qPCR, FISH	ICARDA, Aleppo, Syria
*Ae. tauschii *subsp. *strangulata*	14	DD	K-112	Sequencing, qPCR, FISH	VIR, St.-Petersburg, Russia
*Ae. tauschii *subsp. *typica*	14	DD	K-428	Sequencing	VIR, St.-Petersburg, Russia
*Ae. tauschii* subsp. *tauschii*	14	DD	K-1619	FISH	VIR, St.-Petersburg, Russia
*Triticum aestivum *cv. Chinese Spring	42	BBAADD	–	FISH	–
*Th. bessarabicum*	14	J^b^J^b^	PI 531711	Sequencing, qPCR, FISH	USDA-ARS GRIN

The corrected **Table 1** appears below.

In the published article there were errors in the **Abstract**, page one; the **Introduction**, paragraph two; in **Materials and methods**, *Fluorescense in situ hybridization*, paragraph two; in **Results**, *Fluorescence in situ hybridization*, paragraphs eight, ten, eleven, fifteen, sixteen, and eighteen; in **Discussion,** *Novel markers for chromosome and genome identification*, paragraph seven; in **Discussion,** *Evolutionary changes of repetitive DNA families in Aegilops crassa genome*, paragraph five.

The designation of genomic formulas and chromosomes, the subgenomes of *Aegilops crassa* D^1^ and D^2^ were given incorrectly as D (Abdolmalaki et al., 2019) and D (Adams and Wendel, 2005), respectively.

The corrections have been made throughout the text as follows.

In the **Abstract**, page one, the sentence previously stated:

“It consists of tetraploid (4x) and hexaploid (6x) cytotypes (2*n* = 4x = 28, D^1^D (Abdolmalaki et al., 2019) X^cr^X^cr^ and 2n = 6x = 42, D^1^D (Abdolmalaki et al., 2019) X^cr^X^cr^D^2^D (Adams and Wendel, 2005), respectively) that are similar morphologically.”

The sentence has been corrected as follows:

“It consists of tetraploid (4x) and hexaploid (6x) cytotypes (2*n* = 4x = 28, D^1^D^1^X^cr^X^cr^ and 2n = 6x = 42, D^1^D^1^X^cr^X^cr^D^2^D^2^, respectively) that are similar morphologically.”

In the **Introduction**, paragraph two, the sentence previously stated:

“*Ae*. *crassa* consists of tetraploid and hexaploid cytotypes [2*n* = 4x = 28, D^1^D (Abdolmalaki et al., 2019) X^cr^X^cr^ and 2*n* = 6x = 42, D^1^D (Abdolmalaki et al., 2019) X^cr^X^cr^D (Adams and Wendel, 2005) D (Adams and Wendel, 2005), respectively] that are similar morphologically.”

The sentence has been corrected as follows:

“*Ae*. *crassa* consists of tetraploid and hexaploid cytotypes [2*n* = 4x = 28, D^1^D^1^X^cr^X^cr^ and 2*n* = 6x = 42, D^1^D^1^X^cr^X^cr^D^2^D^2^, respectively] that are similar morphologically.”

In the **Introduction**, paragraph two, the sentence previously stated:

“One of the *Ae*. *crassa* subgenomes, designated D (Abdolmalaki et al., 2019), is related to the D genome of *Ae*. *tauschii* (2*n* = 2x = 14, DD; Kihara, 1949; Kimber and Zhao, 1983; Zhang and Dvořák, 1992; Badaeva et al., 1998, 2002; Edet et al., 2018), which also served as the cytoplasmic genome donor to this tetraploid species (Terachi et al., 1987; Kimber and Tsunewaki, 1988).”

The sentence has been corrected as follows:

“One of the *Ae*. *crassa* subgenomes, designated D^1^, is related to the D genome of *Ae*. *tauschii* (2*n* = 2x = 14, DD; Kihara, 1949; Kimber and Zhao, 1983; Zhang and Dvořák, 1992; Badaeva et al., 1998, 2002; Edet et al., 2018), which also served as the cytoplasmic genome donor to this tetraploid species (Terachi et al., 1987; Kimber and Tsunewaki, 1988).”

In **Materials and methods**, *Fluorescence in situ hybridization*, paragraph two, the sentence previously stated:

“At the same time, we have found earlier that hexaploid *Ae*. *crassa*, IG 131680 carries a translocation T1D (Abdolmalaki et al., 2019)L:7D (Abdolmalaki et al., 2019)L (T10) with interstitial breakpoints in addition to two species-specific translocations, T1 (A^cr^:6X^cr^) and T2 (4D^1^S,F^cr^S; Badaeva et al., 2021).”

The sentence has been corrected as follows:

“At the same time, we have found earlier that hexaploid *Ae*. *crassa*, IG 131680 carries a translocation T1D^1^L:7D^1^L (T10) with interstitial breakpoints in addition to two species-specific translocations, T1 (A^cr^:6X^cr^) and T2 (4D^1^S,F^cr^S; Badaeva et al., 2021).”

In **Results**, *Fluorescence in situ hybridization*, paragraph eight, the sentence previously stated:

“Small CL209-sites are present on chromosomes C^cr^ (middle of the short arm), 5X^cr^ (distal third of the long arm), and on a distal part of 7D (Abdolmalaki et al., 2019)L arm (**Figure 1J**; Supplementary Figures S3, S4).”

The sentence has been corrected as follows:

“Small CL209-sites are present on chromosomes C^cr^ (middle of the short arm), 5X^cr^ (distal third of the long arm), and on a distal part of 7D^1^L arm (**Figure 1J**; Supplementary Figures S3, S4).”

In **Results**, *Fluorescence in situ hybridization*, paragraph eight, the sentence previously stated:

“Hexaploid *Ae*. *crassa* has additional large subtelomeric CL209 clusters on chromosomes 1X^cr^S and in a distal part of 6D (Adams and Wendel, 2005)L (**Figure 2F**; Supplementary Figure S4).”

**Figure 2:**
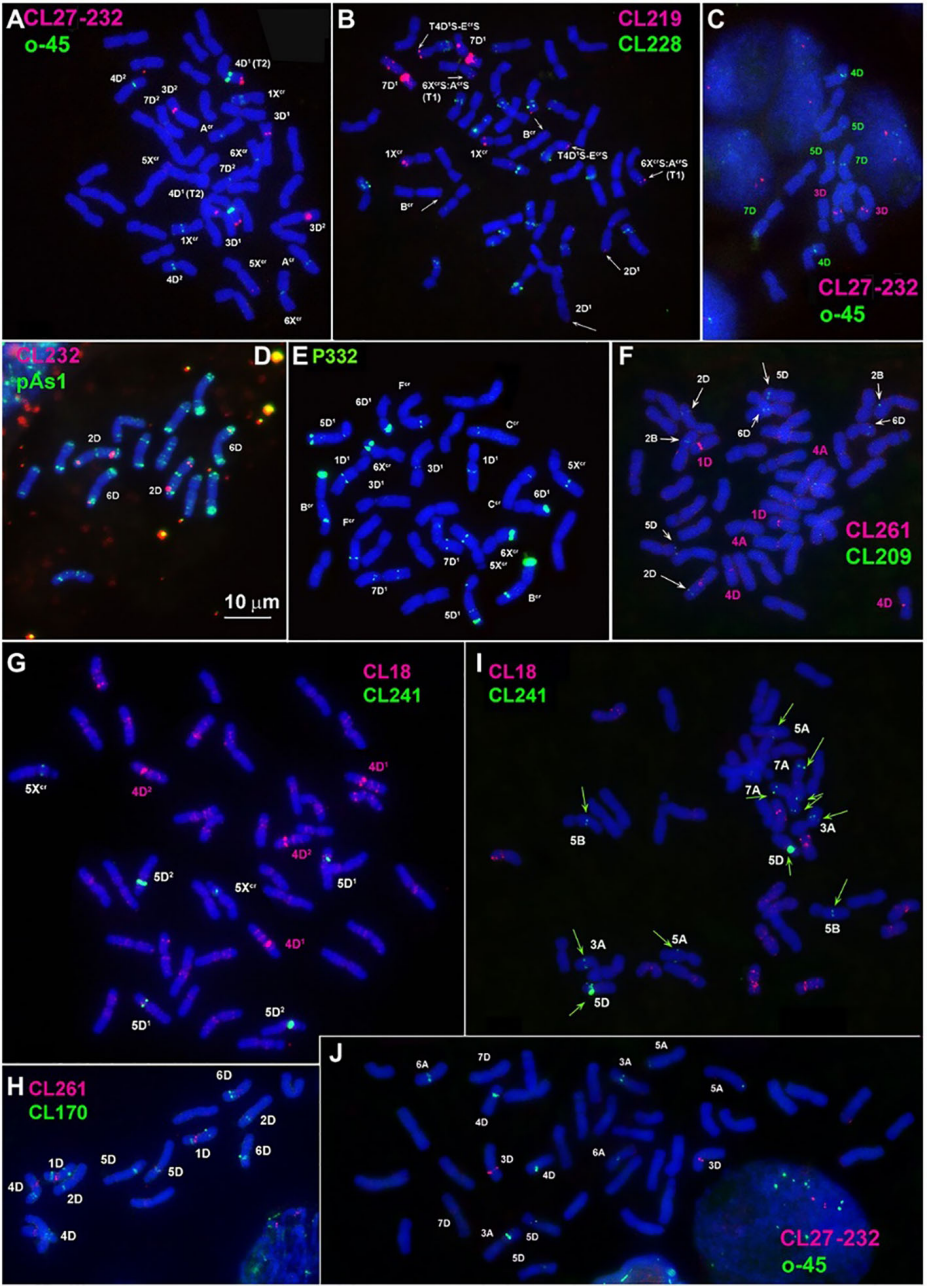
Localization of: **(A, C, J)** CL27_232 (red) + o45 (green), **(B)** CL219 (red) + CL228(green); **(D)** CL232 (red) + pAs1 (green); **(E)** P332 (green); **(F)** CL261(red) + CL209 (green); **(G, I)** CL18 (red) + CL241 (green); **(H)** CL261 (red) + CL170 (green) on chromosomes of *Ae. crassa*, 4x **(E)**, 6x **(A, B, F, G)**, *Ae. tauschii ***(C, D, H)** and common wheat **(I, J)**.

The sentence has been corrected as follows:

“Hexaploid *Ae*. *crassa* has additional large subtelomeric CL209 clusters on chromosomes 1X^cr^S and in a distal part of 6D^2^L (**Figure 2F**; Supplementary Figure S4).”

In **Results**, *Fluorescence in situ hybridization*, paragraph ten, the sentence previously stated:

“Hexaploid *Ae*. *crassa* has smaller signals of CL232 in a terminal part of 4D^1^S and distal quarters of 7D (Abdolmalaki et al., 2019)L and 6D (Adams and Wendel, 2005)L.”

The sentence has been corrected as follows:

“Hexaploid *Ae*. *crassa* has smaller signals of CL232 in a terminal part of 4D^1^S and distal quarters of 7D^1^L and 6D^2^L.”

In **Results**, *Fluorescence in situ hybridization*, paragraph eleven, the sentence previously stated:

“Among them, five sites are common (B^cr^L, C^cr^L, 5X^cr^L, 2D (Abdolmalaki et al., 2019)L, and 3D^1^), but tetraploid form contains two additional loci on 1D (Abdolmalaki et al., 2019)L and 2D^1^S.”

The sentence has been corrected as follows:

“Among them, five sites are common (B^cr^L, C^cr^L, 5X^cr^L, 2D^1^L, and 3D^1^), but tetraploid form contains two additional loci on 1D^1^L and 2D^1^S.”

In **Results**, *Fluorescence in situ hybridization*, paragraph fifteen, the sentence previously stated:

“Two clear signals are detected in the terminus and in the middle part of 1D^1^ and 6D^1^S short arms; a prominent, probably double signal is observed in the terminal part of 3DL (Abdolmalaki et al., 2019), and one or a pair of small signals are present in opposite arms of 2D^1^ and 7D^1^ (**Figure 6**).”

**Figure 6:**
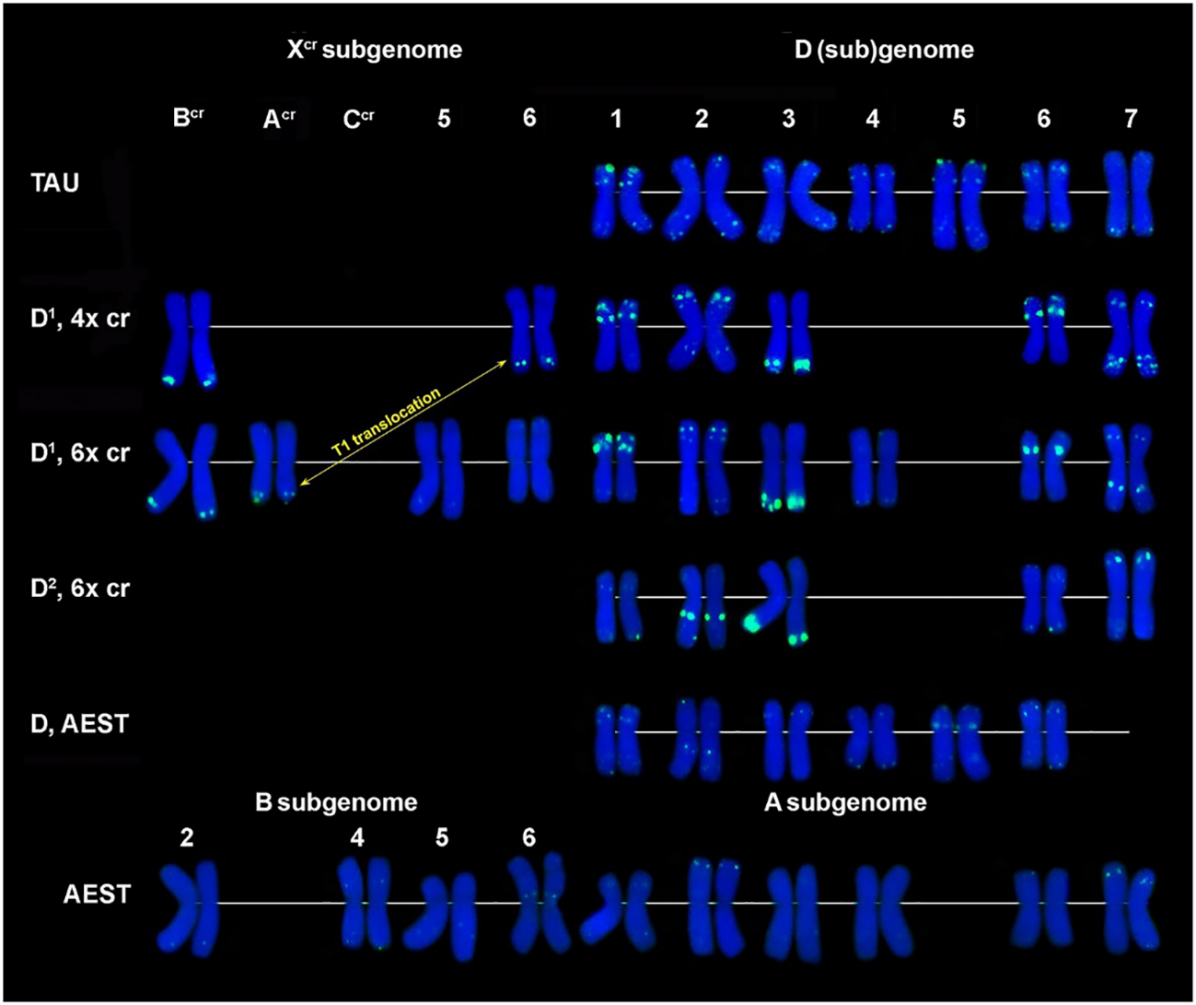
Comparison of CL228 labeling patterns on chromosomes of different cereal species: TAU – *Ae. tauschii *ssp. *strangulata*; D^1^, 4x cr – D^1^ subgenome of tetraploid *Ae. crassa*; D^1^, 6x cr, D^2^, 6x cr – D^1^ and D^2^ subgenomes of hexaploid *Ae. crassa*; AEST – A, B, and D subgenomes of *T. aestivum*. 1–7 – homoeologous groups. White arrowheads show minor CL228 sites detected on the X^cr^, A, B, and D subgenome chromosomes.

The sentence has been corrected as follows:

“Two clear signals are detected in the terminus and in the middle part of 1D^1^ and 6D^1^ short arms; a prominent, probably double signal is observed in the terminal part of 3D^1^L, and one or a pair of small signals are present in opposite arms of 2D^1^ and 7D^1^ (**Figure 6**).”

In **Results**, *Fluorescence in situ hybridization*, paragraph sixteen, the sentence previously stated:

“The largest signal occurs on 3DL (Adams and Wendel, 2005), similarly to 3DL (Abdolmalaki et al., 2019), and other intense sites are present in the proximal third of 2DL (Adams and Wendel, 2005) and a distal part of 7D^2^S.”

The sentence has been corrected as follows:

“The largest signal occurs on 3D^2^L, similarly to 3D^1^L, and other intense sites are present in the proximal third of 2D^2^L and a distal part of 7D^2^S.”

In **Results**, *Fluorescence in situ hybridization*, paragraph eighteen, the sentence previously stated “Most prominent sites appear on chromosome 2D^1^; subterminal signals on 3DL (Abdolmalaki et al., 2019) and 3DL (Adams and Wendel, 2005) are also large.”

The sentence has been corrected as follows:

“Most prominent sites appear on chromosome 2D^1^; subterminal signals on 3D^1^L and 3D^2^L are also large.”

In **Discussion**, *Novel markers for chromosome and genome identification*, paragraph seven, the sentence previously stated:

“Thus, three prominent CL131 clusters were detected on 2D^1^ and another one, overlapping with CL228, on 3DL (Abdolmalaki et al., 2019).”

The sentence has been corrected as follows:

“Thus, three prominent CL131 clusters were detected on 2D^1^ and another one, overlapping with CL228, on 3D^1^L.”

In **Discussion**, *Evolutionary changes of repetitive DNA families in Aegilops crassa genome*, paragraph five, the sentence previously stated:

“Genomic shock might cause massive amplification and spread of the repeat to other chromosomal sites, leading to the emergence of prominent CL219 clusters in proximal (7D^1^S) and distal 7DL (Abdolmalaki et al., 2019) chromosome regions.”

The sentence has been corrected as follows:

“Genomic shock might cause massive amplification and spread of the repeat to other chromosomal sites, leading to the emergence of prominent CL219 clusters in proximal (7D^1^S) and distal (7D^1^L) chromosome regions.”

In the **Introduction**, paragraph two, a sentence previously stated:

“The D subgenome is also present in two polyploid *Aegilops* species and in four exaploidy bread wheat *Triticum aestivum* (2n = 6x = BBAADD).”

The sentence has been corrected as follows:

“The D subgenome is also present in two polyploid *Aegilops* species and in hexaploid bread wheat *Triticum aestivum* (2n = 6x = BBAADD).”

In the **Introduction**, paragraph two, a sentence previously stated:

“According to molecular analysis of nuclear genome (Dvořák et al., 1998; Luo et al., 2017; Singh et al., 2019) and hybridization pattern of pAs1 probe (Badaeva et al., 1996, 2019a; Zhao et al., 2018; Ebrahimzadegan et al., 2021), the D subgenome of polyploid wheat was inherited from *Ae*. *tauschii* subsp. four exaploidy, while *Ae*. *tauschii* subsp. *tauschii* contributed the D subgenome to polyploid *Aegilops* species *Ae*. *cylindrica* and 6x *Ae*. *crassa* (Badaeva et al., 2002).”

The sentence has been corrected as follows:

“According to molecular analysis of nuclear genome (Dvořák et al., 1998; Luo et al., 2017; Singh et al., 2019) and hybridization pattern of pAs1 probe (Badaeva et al., 1996, 2019a; Zhao et al., 2018; Ebrahimzadegan et al., 2021), the D subgenome of polyploid wheat was inherited from *Ae*. *tauschii* subsp. *strangulata*, while *Ae*. *tauschii* subsp. *tauschii* contributed the D subgenome to polyploid *Aegilops* species *Ae*. *cylindrica* and 6x *Ae*. *crassa* (Badaeva et al., 2002).”

In the **Introduction**, paragraph three, a sentence previously stated:

“Kihara (1963) proposed that this subgenome could be inherited from *Ae*. *comosa* and suggested genomic formula DM for tetraploid and DDM for four hexaploid *Ae*. *crassa*.”

The sentence has been corrected as follows:

“Kihara (1963) proposed that this subgenome could be inherited from *Ae*. *comosa* and suggested genomic formula DM for tetraploid and DDM for hexaploid *Ae*. *crassa*.”

In **Materials and methods**, *Real-time quantitative PCR*, paragraph one, a sentence previously stated:

“Primers 2) were synthesized at Syntol Ltd. (Moscow, Russia).”

The sentence has been corrected as follows:

“Primers were synthesized at Syntol Ltd. (Moscow, Russia).”

In **Materials and methods**, *DNA probes for FISH*, paragraph one, a sentence previously stated:

“The following novel repeats identified by means of low-coverage sequencing followed by bioinformatics analysis were used as FISH probes: (i) derived from 4× *Ae. crassa* genome: CL3, CL8 (highly homologous to CL16 found in Ae. Tauschii subsp. nine trangulate), CL18, CL60, CL131 (highly homologous to CL149 found in *Th*. *bessarabicum*), CL170, CL193, CL209, CL219, CL228, CL232, CL239, CL241, CL244, CL257, CL258, CL261 (highly homologous to CL198 found in *Th*. *bessarabicum*); ii) derived from 6x *Ae. crassa* genome: CL27_232; iii) derived from *Th*. *bessarabicum* genome: CL2, CL148.”

The sentence has been corrected as follows:

“The following novel repeats identified by means of low-coverage sequencing followed by bioinformatics analysis were used as FISH probes: (i) derived from 4× *Ae. crassa* genome: CL3, CL8 (highly homologous to CL16 found in *Ae. tauschii* subsp. *strangulata*), CL18, CL60, CL131 (highly homologous to CL149 found in *Th*. *bessarabicum*), CL170, CL193, CL209, CL219, CL228, CL232, CL239, CL241, CL244, CL257, CL258, CL261 (highly homologous to CL198 found in *Th*. *bessarabicum*); ii) derived from 6x *Ae. crassa* genome: CL27_232; iii) derived from *Th*. *bessarabicum* genome: CL2, CL148.”

In **Materials and methods**, *Fluorescence in situ hybridization*, paragraph one, a sentence previously stated:

“The A subgenome chromosomes of wheat were classified using additional probe combination GAA_n_ and pTa-535 according to Komuro et al. (2013); chromosomes of *Ae*. *Tauschii* were classified as suggested by Badaeva et al. (2002, 2019a) and Zhao et al. (2018), *Ae*. *crassa* as in Abdolmalaki et al. (2019), and Badaeva et al. (2021).”

The sentence has been corrected as follows:

“The A subgenome chromosomes of wheat were classified using additional probe combination GAA_n_ and pTa-535 according to Komuro et al. (2013); chromosomes of *Ae*. *tauschii* were classified as suggested by Badaeva et al. (2002, 2019a) and Zhao et al. (2018), *Ae*. *crassa* as in Abdolmalaki et al. (2019), and Badaeva et al. (2021).”

In **Materials and methods**, *Fluorescence in situ hybridization*, paragraph two, the sentence previously stated:

“Our previous analyses showed that accessions K-2485 and AE 742 (*Ae*. *crassa*, 4x), K-112 (*Ae*. *tauschii* subsp. *strangulata*), PI 201890 (*Th*. *bessarabicum*), and Chinese Spring have normal karyotypes typical to the respective species (Gill et al., 1991; Badaeva et al., 2019a, 2019b, 2021).”

The sentence has been corrected as follows:

“Our previous analyses showed that accessions K-2485 and AE 742 (*Ae*. *crassa*, 4x), K-112 (*Ae*. *tauschii* subsp. *strangulata*), PI 531711 (*Th*. *bessarabicum*), and Chinese Spring have normal karyotypes typical to the respective species (Gill et al., 1991; Badaeva et al., 2019a, 2019b, 2021).”

In **Results**, *Repeats’ characterization*, paragraph two, point one previously stated: “AC4x_CL3_339nt was more dissimilar to P335 repeat from *Th*. *besarabicum* genome.”

This has been corrected as follows:

“AC4x_CL3_339nt was more dissimilar to P335 repeat from *Ae. tauschii* genome.”

In **Results**, *Fluorescence in situ hybridization*, paragraph seventeen, a sentence previously stated:

“Several very weak but consistent signals appear on 1B, 4B, 5B, 1A, 3A, 4A, and 6A chromosomes (**Figure 6**).”

The sentence has been corrected as follows:

“Several very weak but consistent signals appear on 2B, 4B, 5B, 1A, 3A, 4A, and 6A chromosomes (**Figure 6**).”

In **Results**, *Fluorescence in situ hybridization*, paragraph twenty-one, a sentence previously stated:

“For all these repeats heteromorphisms of homologous chromosomes in signal presence and/ or size is often observed.”

The sentence has been corrected as follows:

“For all these repeats heteromorphisms of homologous chromosomes in signal presence and/ or size is often observed.”

In **Discussion**, *Novel markers for chromosome and genome identification*, paragraph nine, a sentence previously stated:

“According to bioinformatics and qPCR the novel CL239 repeat is absent in *Ae*. *tauschii* (sub)genomes.”

The sentence has been corrected as follows:

“According to bioinformatics and qPCR the novel CL239 repeat is absent in the *Ae*. *tauschii* genome.”

In **Discussion**, *Evolutionary changes of repetitive DNA families in Aegilops crassa genome*, paragraph eight, a sentence previously stated:

“We can reach a similar conclusion considering that the CL170 repeat is absent from X^cr^ genome, but is abundant in the D (sub)genome of wheat and *Ae*. *tauschii*, *Ae*. *crassa*, and *Th*. *bessarabicum*.”

The sentence has been corrected as follows:

“We can reach a similar conclusion considering that the CL170 repeat is absent in X^cr^ genome, but is abundant in the D (sub)genome of wheat and *Ae*. *tauschii*, *Ae*. *crassa*, and *Th*. *bessarabicum*.”

The publisher apologizes for these errors. The original article has been updated.

