# Supplementary figures and images for: Aegilops crassa Boiss. repeatome characterized using low-coverage NGS as a source of new FISH markers: Application in phylogenetic studies of the Triticeae
Source: Front Plant Sci. 2022 Oct 5;13:980764. doi: 10.3389/fpls.2022.980764 (PMC9621091; doi:10.3389/fpls.2022.980764)

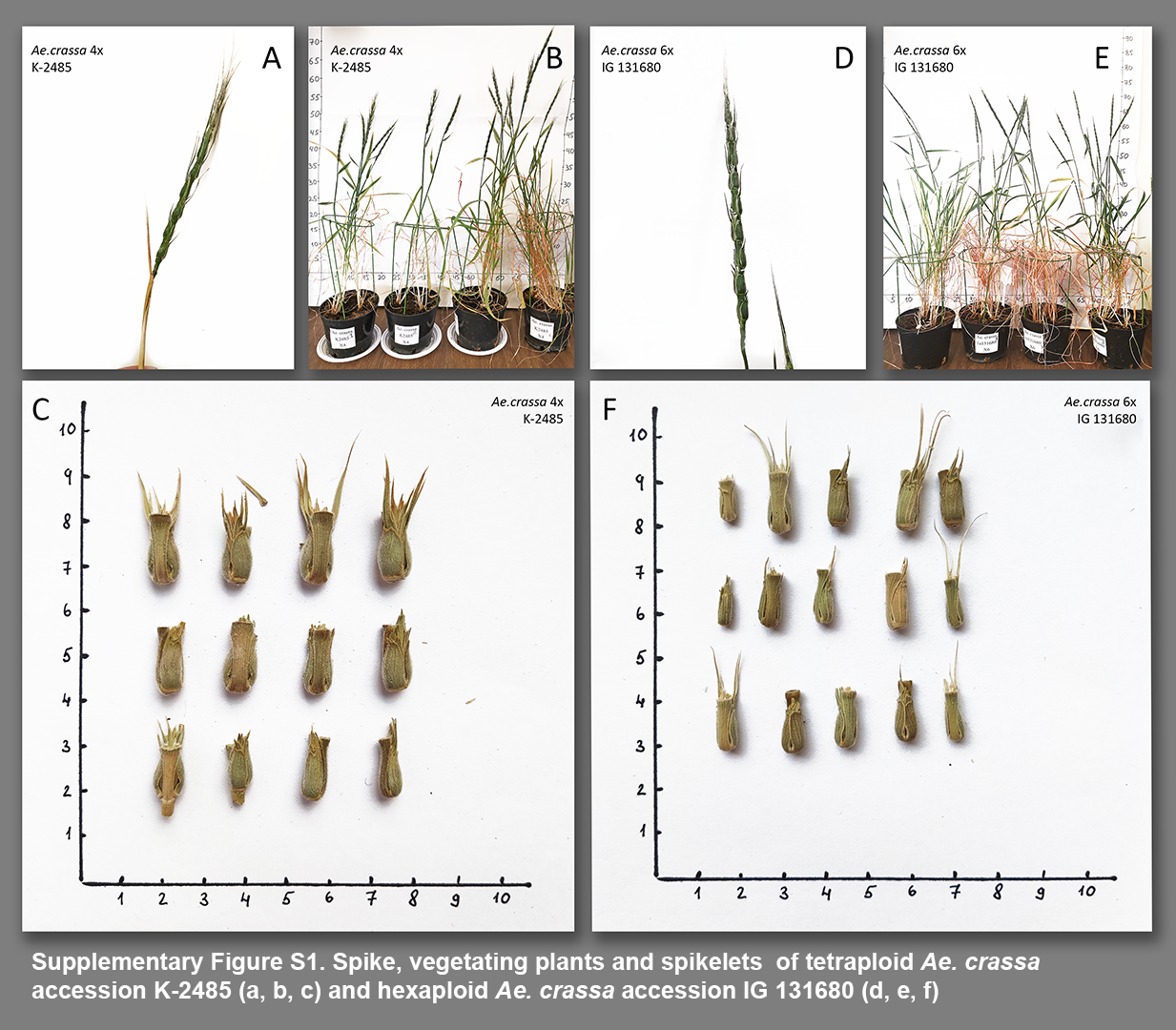

Supplement: Supplementary file 1 [file Image_1.TIF]

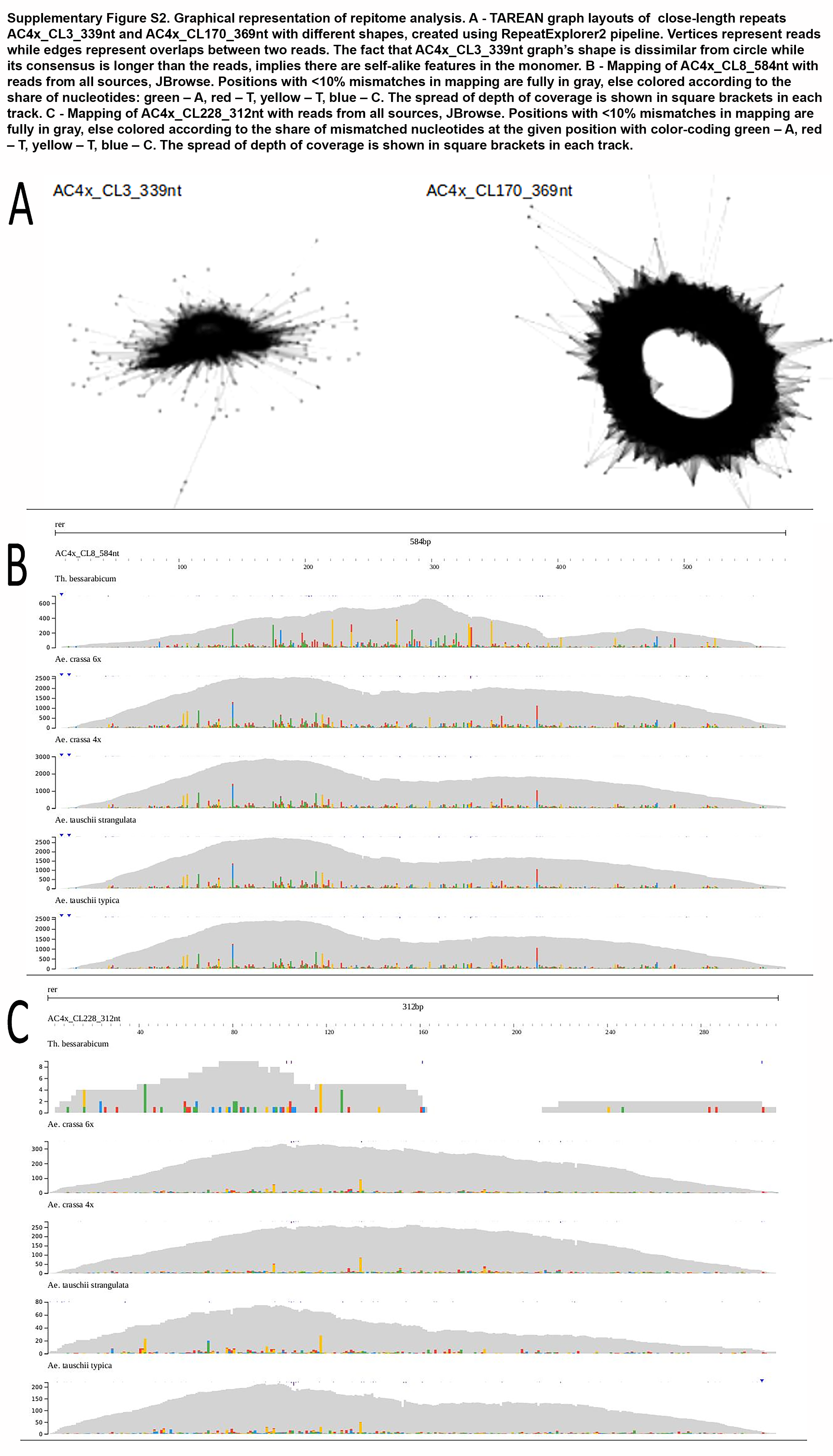

Supplement: Supplementary file 2 [file Image_2.TIF]

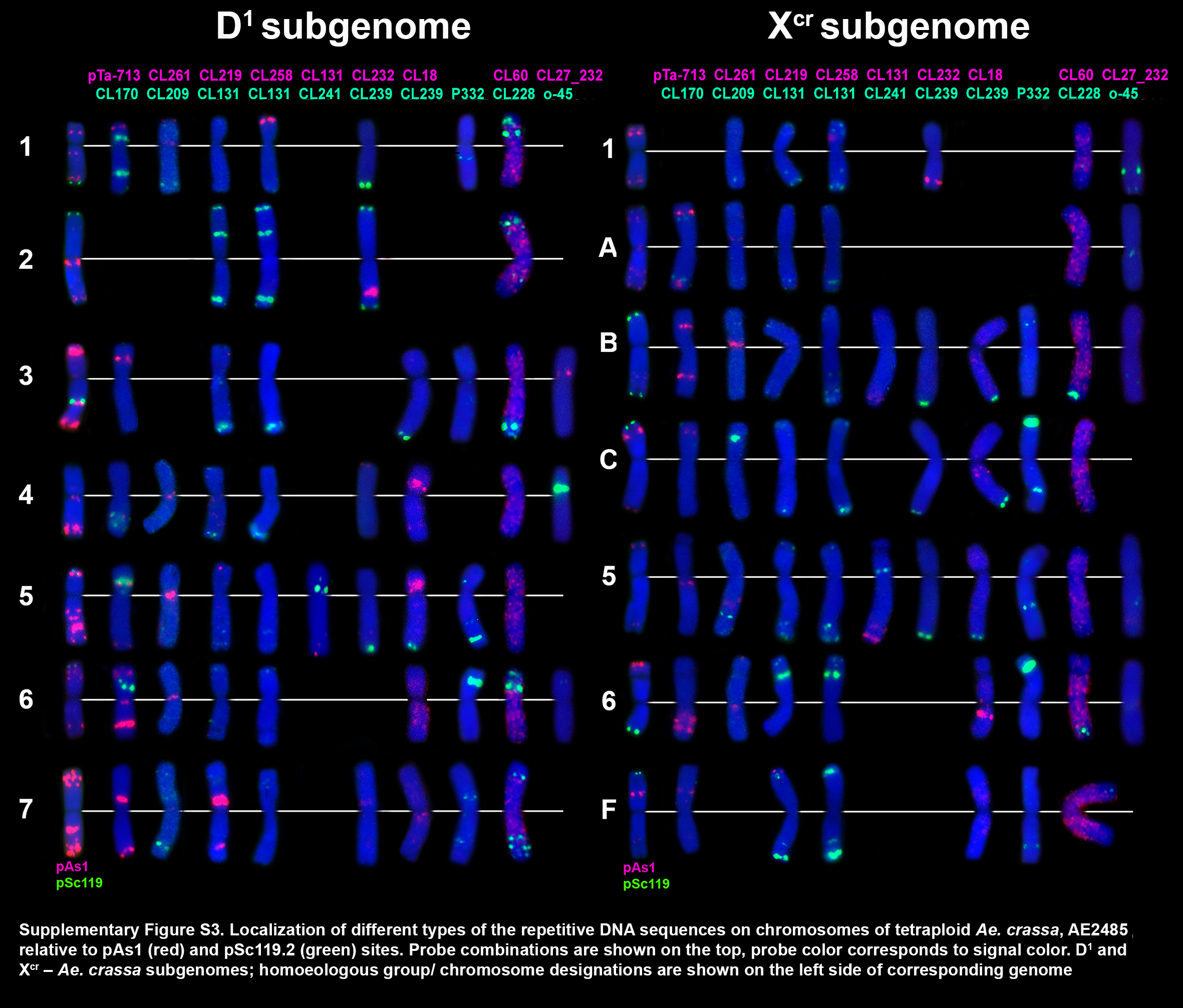

Supplement: Supplementary file 3 [file Image_3.TIF]

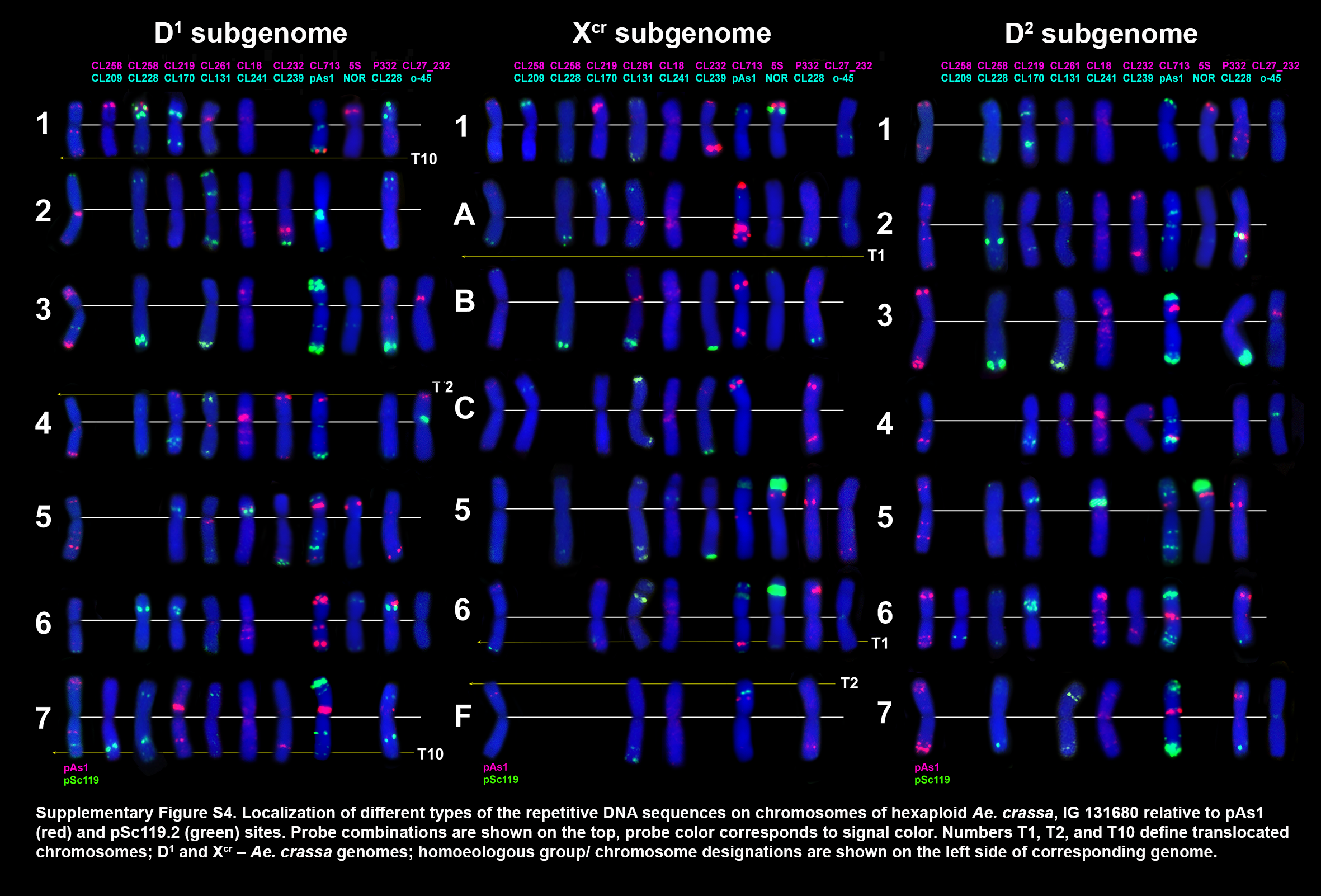

Supplement: Supplementary file 4 [file Image_4.TIF]

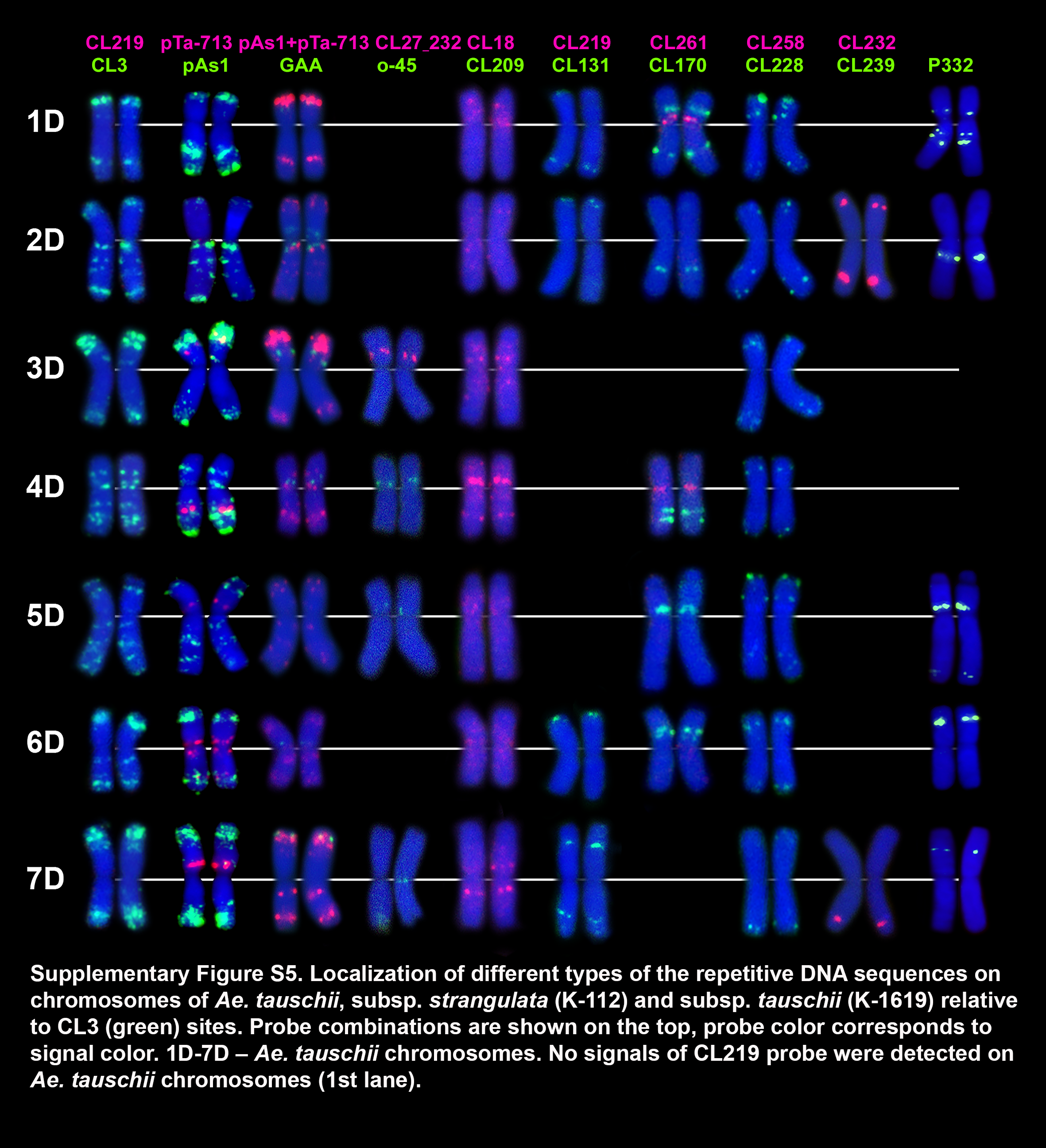

Supplement: Supplementary file 5 [file Image_5.TIF]

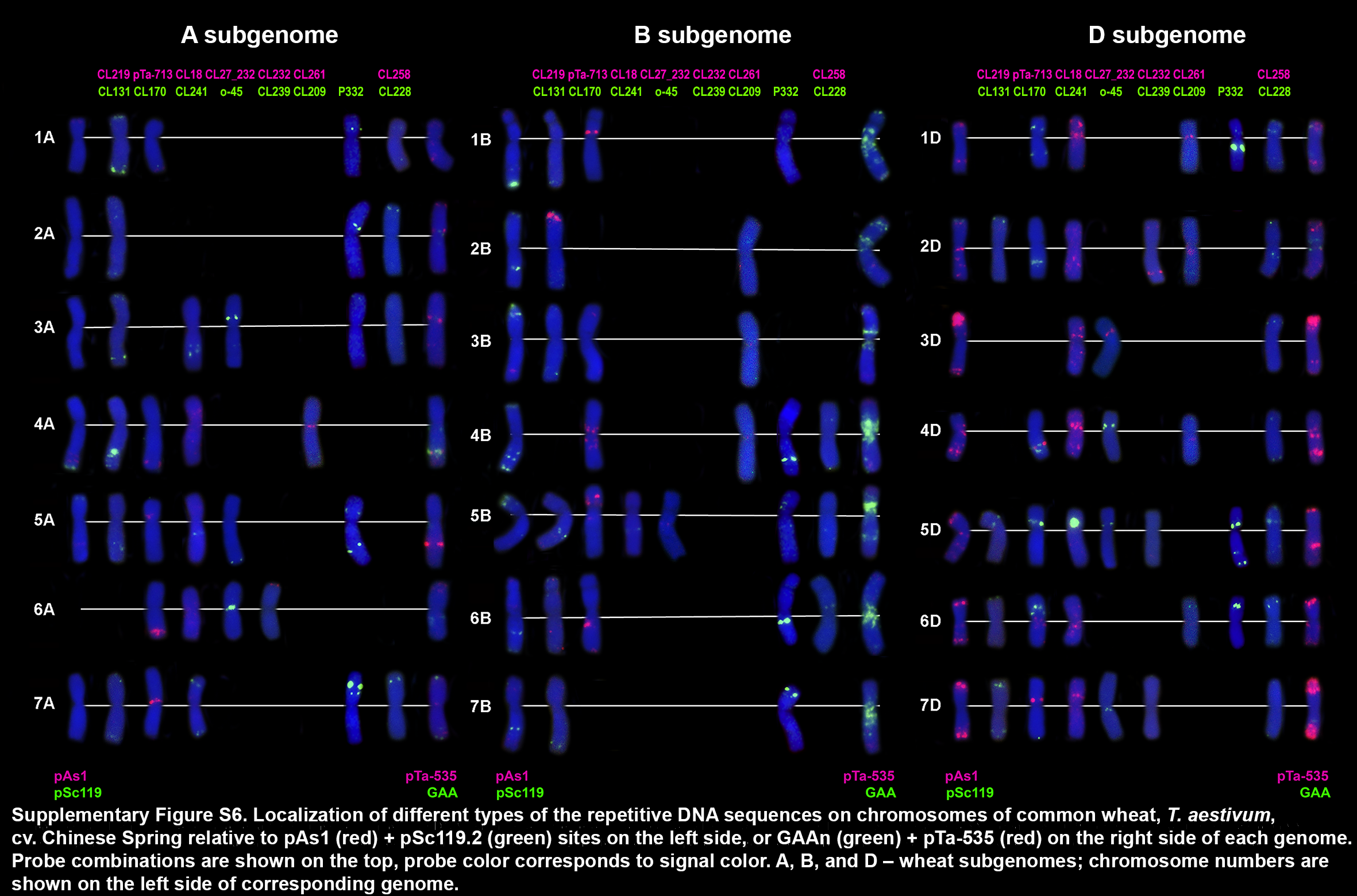

Supplement: Supplementary file 6 [file Image_6.TIF]

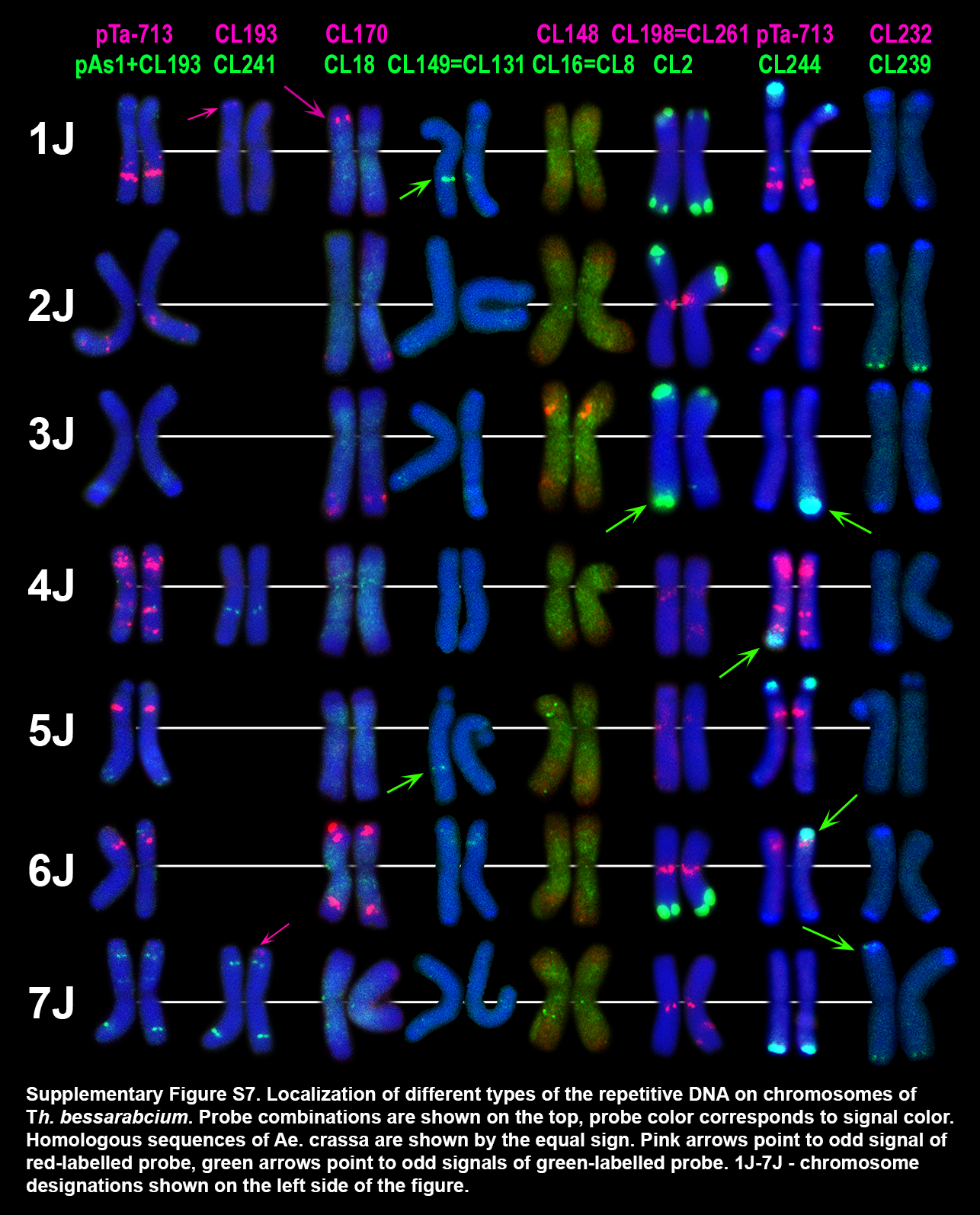

Supplement: Supplementary file 7 [file Image_7.TIF]
